# Evaluation of Genome Wide Association Study Associated Type 2 Diabetes Susceptibility Loci in Sub Saharan Africans

**DOI:** 10.3389/fgene.2015.00335

**Published:** 2015-11-24

**Authors:** Adebowale A. Adeyemo, Fasil Tekola-Ayele, Ayo P. Doumatey, Amy R. Bentley, Guanjie Chen, Hanxia Huang, Jie Zhou, Daniel Shriner, Olufemi Fasanmade, Godfrey Okafor, Benjamin Eghan, Kofi Agyenim-Boateng, Jokotade Adeleye, Williams Balogun, Abdel Elkahloun, Settara Chandrasekharappa, Samuel Owusu, Albert Amoah, Joseph Acheampong, Thomas Johnson, Johnnie Oli, Clement Adebamowo, Francis Collins, Georgia Dunston, Charles N. Rotimi

**Affiliations:** ^1^Center for Research on Genomics and Global Health, National Human Genome Research Institute, National Institutes of HealthBethesda, MD, USA; ^2^Department of Medicine, University of LagosLagos, Nigeria; ^3^Department of Medicine, University of Nigeria Teaching HospitalEnugu, Nigeria; ^4^Department of Medicine, University of Science and TechnologyKumasi, Ghana; ^5^Department of Medicine, College of Medicine, University of IbadanIbadan, Nigeria; ^6^National Human Genome Research Institute, National Institutes of HealthBethesda, MD, USA; ^7^Department of Medicine and Therapeutics, University of Ghana Medical SchoolAccra, Ghana; ^8^Institute of Human Virology, School of Medicine, University of MarylandBaltimore, MD, USA; ^9^National Institutes of HealthBethesda, MD, USA; ^10^National Human Genome Center at Howard UniversityWashington, DC, USA

**Keywords:** genetic association, replication, fine-mapping, type 2 diabetes, sub Saharan Africa

## Abstract

Genome wide association studies (GWAS) for type 2 diabetes (T2D) undertaken in European and Asian ancestry populations have yielded dozens of robustly associated loci. However, the genomics of T2D remains largely understudied in sub-Saharan Africa (SSA), where rates of T2D are increasing dramatically and where the environmental background is quite different than in these previous studies. Here, we evaluate 106 reported T2D GWAS loci in continental Africans. We tested each of these SNPs, and SNPs in linkage disequilibrium (LD) with these index SNPs, for an association with T2D in order to assess transferability and to fine map the loci leveraging the generally reduced LD of African genomes. The study included 1775 unrelated Africans (1035 T2D cases, 740 controls; mean age 54 years; 59% female) enrolled in Nigeria, Ghana, and Kenya as part of the Africa America Diabetes Mellitus (AADM) study. All samples were genotyped on the Affymetrix Axiom PanAFR SNP array. Forty-one of the tested loci showed transferability to this African sample (*p* < 0.05, same direction of effect), 11 at the exact reported SNP and 30 others at SNPs in LD with the reported SNP (after adjustment for the number of tested SNPs). *TCF7L2* SNP rs7903146 was the most significant locus in this study (*p* = 1.61 × 10^−8^). Most of the loci that showed transferability were successfully fine-mapped, i.e., localized to smaller haplotypes than in the original reports. The findings indicate that the genetic architecture of T2D in SSA is characterized by several risk loci shared with non-African ancestral populations and that data from African populations may facilitate fine mapping of risk loci. The study provides an important resource for meta-analysis of African ancestry populations and transferability of novel loci.

## Introduction

Sub-Saharan Africa (SSA) is one of the regions with the fastest growth in type 2 diabetes (T2D) worldwide (Wild et al., 2004). There are an estimated 19.8 million people with type 2 diabetes in SSA in 2013 and this number is projected to increase to 41.5 million by the year 2035 (IDF, 2013). Genome-wide association studies (GWAS) have been particularly productive for understanding the genetic basis of T2D, with over 100 associated susceptibility loci reported, including a recent large meta-analysis (n ~150,000) yielding 65 loci (Morris et al., 2012) in European ancestry populations alone. However, most of these success stories have come from European and Asian ancestry populations. A few GWAS for T2D have been done in African Americans, including a meta-analysis (Ng et al., 2014), but there is currently no similar study of indigenous Africans. To date, only one genome-wide linkage study of T2D in an African population has been published (Rotimi et al., 2004) and a GWAS of T2D in a SSA population has not yet been done. Here, we report a replication and fine mapping analysis of T2D in SSA with 1775 subjects (1035 cases, 740 controls) genotyped on the Affymetrix Axiom® PanAFR array (imputed into the 1000 Genomes phase 1 v3 reference panel). Given the relatively modest sample size and limited power for novel discovery, we focus on evaluation of previously reported T2D GWAS loci in this study of indigenous Africans, including looking for evidence of replication or transferability and conducting fine mapping studies to test whether the relatively weaker linkage disequilibrium (LD) and smaller haplotypes in this African sample could improve the resolution of previously reported loci.

## Materials and methods

### Ethics statement

Ethical approval for the study was obtained from the Institutional Review Board (IRB) of each participating institution. All subjects provided written informed consent for the collection of samples and subsequent analysis. This study was conducted in accordance with the principles expressed in the Declaration of Helsinki.

### Study participants

The initial study sample consisted of 1822 unrelated subjects from the Africa America Diabetes Mellitus (AADM) study (Rotimi et al., 2001, 2004), a genetic epidemiology study of T2D in SSA. All subjects were SSA, enrolled from university medical centers in Nigeria, Ghana, and Kenya. Patients attending medical clinics at these medical centers or patients referred for clinical suspicion of diabetes were evaluated for potential inclusion in the study as described below. After providing informed consent, all participants underwent a clinical examination that included a medical history, clinical anthropometry, blood pressure measurements and blood sampling. Weight was measured in light clothes on an electronic scale to the nearest 0.1 kg, and height was measured with a stadiometer to the nearest 0.1 cm. Body mass index (BMI) was computed as weight in kg divided by the square of the height in meters. The other clinical measurements have been described elsewhere (Rotimi et al., 2001, 2004). The definition of T2D was based on the American Diabetes Association (ADA) criteria: a fasting plasma glucose concentration (FPG) ≥ 126 mg/dl (7.0 mmol/l) or a 2-h postload value in the oral glucose tolerance test ≥ 200 mg/dl (11.1 mmol/l) on more than one occasion. Alternatively, a diagnosis of T2D was accepted if an individual was on pharmacological treatment for T2D and review of clinical records indicated adequate justification for that therapy. The detection of autoantibodies to glutamic acid decarboxylase (GAD) and/or a fasting C-peptide ≤ 0.03 nmol/l was used to exclude probable cases of type 1 diabetes. Controls were required to have FPG < 110 mg/dl or 2-h postload of < 140 mg/dl and no symptoms suggestive of diabetes (the classical symptoms being polyuria, polydipsia, and unexplained weight loss).

### Genotyping

Samples were genotyped on the Affymetrix Axiom® PANAFR SNP array. This array of ~2.1 million SNPs is one of Affymetrix's Axiom® Genome-Wide Population-Optimized Human Arrays and is optimized for African ancestry populations. The array offers pan-African genomic coverage, with ≥90% genetic coverage of common and rare variants (MAF >2%) of the Yoruba (West African) genome and >85% coverage of common and rare variants (MAF >2%) of the Luhya and Maasai (East African) genomes. Starting from 1822 subjects, 14 (one duplicated and 13 sex-discordant) samples were excluded after initial quality control and 33 subjects were excluded because they showed cryptic relatedness with other subjects (IBD Pi^∧^Hat > 0.125 indicating more than 3rd degree relatedness). The remaining 1775 subjects (1035 T2D cases, 740 controls) formed the basis of this analysis. The sample-level genotype call rate was at least 0.95 for all subjects. The 1775 subjects included 1598 (90%) West Africans enrolled from Nigeria and Ghana (Rotimi et al., 2001, 2004) and 177 (10%) East Africans enrolled from Kenya. The most common ethnic groups represented were: Yoruba (31.2%), Igbo (23.5%), Akan (20.5%), Gaa-Adangbe (8.6%), and Kalenjin (5.6%).

The initial set of 2,217,748 SNPs was filtered for missingness, Hardy-Weinberg equilibrium (HWE) and allele frequency as described in Supplementary Table 1. SNP level filters that were applied included: missingness > 0.05 (*n* = 94, 438), HWE *p* < 1 × 10^−6^ (*n* = 20, 472) and minor allele frequency < 0.01 (*n* = 45, 759). The allele frequency spectrum of the SNPs that passed QC is shown in Supplementary Figure 1. SNPs that passed quality control were used as the basis for imputation. The samples clustered as expected (Supplementary Figure 2) based on principal components (PCs) of the genotypes computed using an LD-pruned subset of 140,000 autosomal SNPs. Imputation was done with the MaCH - http://www.sph.umich.edu/csg/abecasis/MACH/index.html (Li et al., 2010)/MaCH-ADMIX—http://www.unc.edu/~yunmli/MaCH-Admix/ (Liu et al., 2013) programs using the 1000 Genomes Consortium phase 1, version 3 cosmopolitan reference filtered for monomorphic and singleton sites (ftp://share.sph.umich.edu/1000genomes/fullProject/2012.03.14/GIANT.phase1_release_v3.20101123.snps_indels_svs.genotypes.refpanel.ALL.vcf.gz.tgz). The resulting imputed dosage data were filtered for imputed allelic dosage frequency < 0.01 and *r*^2^ < 0.3, yielding ~15M SNPs for analysis.

### Statistical analysis

Association analysis was done with *mach2dat* using the imputed SNP dosage data within a logistic regression framework. Use of the allelic dosages is preferable to using the best guess genotypes because it accounts for the uncertainty in imputation. Covariates included were age, sex, BMI and the first three PCs of the genotypes. Residual population stratification was low (genomic inflation factor, λ = 1.016) after adjusting for the first three PCs of the genotypes (Supplementary Figure 3). Top association hits with a *p* ≤ 5 × 10^−7^ can be found in the Supplementary Tables.

We looked for evidence of transferability of established T2D susceptibility loci reported in the literature from GWAS and meta-analysis of GWAS (*n* = 106 loci—Supplementary Table 2). More than half of these susceptibility loci (*n* = 65) were reported in the largest meta-analysis of T2D in populations of European ancestry (DIAGRAM+) (Morris et al., 2012). We first examined the *p*-value at each reported SNP in our study (exact transferability or replication) and considered a *p* < 0.05 and consistency of direction of effect for the same allele as evidence for significant transferability. Next, we examined all the SNPs in the LD block (as determined by the method of Gabriel et al., 2002) containing the index SNP in the 1000 Genomes EUR or CHB population reference (as appropriate for the discovery hit) for evidence of local transferability. *P*-values were adjusted for the number of SNPs tested around each index SNP. The number of independent SNPs was determined and correction for multiple testing was done using the method of the effective degrees of freedom for the spectrally decomposed covariance matrix for the block of SNPs (Bretherton et al., 1999; Ramos et al., 2011). Briefly, we estimate the covariance matrix for the block of SNPs using the genotype data. Then, the covariance matrix was spectrally decomposed and the effective degrees of freedom (*N*_*eff*_) estimated using the relationship, $N_{eff}={\left(\sum_{K=1}^{K}\lambda_{k}\right)}^{2}/\left(\sum_{K=1}^{K}\lambda_{k}^{2}\right)$, in which λ_*k*_ is the kth eigenvalue of the *K*×*K* covariance matrix for the K SNPs. Finally, the nominal significance threshold α = 0.05 was divided by *N*_*eff*_. We consider the “best SNP” in the haplotype block as the SNP showing the smallest *p*-value and that is in LD with the reported SNP. Using data from our study sample and from the 1000 Genomes YRI, haplotype blocks were constructed around each locus that showed transferability to determine if African ancestry samples helped to fine-map the locus.

The original reports of the loci studied presented effect sizes ranging from an OR of 1.01–1.6 in most studies and the minor allele frequencies at the risk loci ranged from 0.02 to 0.49 in our dataset. We estimated power for replication of a reported SNP at a one-sided α of 0.05 (i.e., same direction of effect) for OR ranging from 1.10 to 1.50 and for a range of allele frequencies in our data set (Supplementary Figure 4). For example, power for replication was 83% for a locus with OR 1.2 at a risk allele frequency of 0.2 and 84% for a locus with OR 1.3 at a risk allele frequency of 0.1. In contrast, at a risk allele frequency of 0.02, our power for replication exceeded 70% only for loci with OR = 1.6.

## Results

The characteristics of the study participants are shown in Table 1. The mean age was 53.8 years and mean BMI was 26.3 kg/m^2^. Participants with T2D had a mean waist circumference that was ~4 cm larger than that of controls (Table 1). The mean fasting glucose of the subjects with T2D [178.8 (SE 2.9) mg/dl] indicates that most subjects had poorly controlled glycemic status on enrollment.

**Table 1:** **Characteristics of subjects**.

**Characteristic**	**Type 2 diabetes cases**	**Controls**
N	1035	740
Sex (% Female)	57.6	61.0
Age (years)	55.4 (0.3)	52.0 (0.4)
Body mass index (BMI) kg/m^2^	26.6 (0.2)	26.1 (0.2)
Waist circumference (cm)	93.9 (0.4)	90.0 (0.4)
Hypertension (%)	61.9	51.6
Fasting glucose (mg/dl)	178.8 (2.9)	87.4 (0.4)
Fasting cholesterol (mg/dl)	208.3 (1.9)	204.2 (2.1)
Fasting triglycerides (mg/dl)	121.7 (2.2)	100.3 (1.8)

*All figures are mean (SE) except where otherwise indicated*.

### Transferability of reported GWAS type 2 diabetes susceptibility loci

From our association tests, we looked for evidence of transferability of 106 established T2D SNPs. We had data on 103 of the 106 SNPs in our dataset. We found exact replication with the index SNP (same allele, consistent direction of effect, *p* < 0.05) with 11 loci (Table 2). Using a local replication strategy in which we examined SNPs in LD with the reported index SNP, we found an additional 30 SNPs showing significant association with T2D in this dataset (Table 3). In sum, we found significant association with T2D for 41 of the 103 GWAS established T2D loci we examined in this study. Overall, 76 of the 103 tested SNPs are directionally consistent with the initial report (*p* = 1 × 10^−6^, binomial test). The *TCF7L2* SNP rs7903146 showed the strongest association with T2D in this study (*p* = 1.61 × 10^−8^, OR 1.50, 95% CI 1.26–2.15)—Supplementary Table 5. It should be noted that this SNP shows the strongest evidence of association with T2D in most GWAS and remains the most consistently associated locus in most populations studied so far.

**Table 2:** **Reported GWAS associated SNPs showing exact transferability in the AADM Study**.

**SNP**	**Chr**	**BP**	**Gene**	**A1/A2**	**A1 Freq**	**β**	***SE*(β)**	**P**
rs7903146	10	114758349	*TCF7L2*	C/T	0.672	−0.466	0.083	1.52E−08
rs1470579	3	185529080	*IGF2BP2*	A/C	0.132	−0.349	0.101	5.09E−04
rs3786897	19	33893008	*PEPD*	A/G	0.392	0.238	0.073	1.15E−03
rs3802177	8	118185025	*SLC30A8*	G/A	0.961	0.582	0.195	2.77E−03
rs4457053	5	76424949	*ZBED3*	G/A	0.162	0.276	0.101	6.03E−03
rs12304921	12	51357542	*HIGD1C*	A/G	0.830	−0.247	0.096	0.010
rs13389219	2	165528876	*GRB14*	C/T	0.216	0.221	0.094	0.019
rs11642841	16	53845487	*FTO*	C/A	0.945	−0.413	0.184	0.023
rs7177055	15	77832762	*HMG20A*	G/A	0.728	−0.174	0.080	0.029
rs972283	7	130466854	*KLF14*	A/G	0.087	−0.274	0.131	0.037
rs10440833	6	20688121	*CDKAL1*	T/A	0.772	−0.178	0.088	0.042

*Base-pair positions are in NCBI build 37 coordinates*.

**Table 3:** **GWAS associated SNPs showing local transferability in the AADM Study**.

**SNP**	**Genotyped (G)/Imputed (I)**	**Imputation *r*^2^**	**Gene**	**Chr**	**BP**	**A1**	**A2**	**Freq A1**	**β**	***SE*(β)**	^*^P_adj_	**Reported SNP**	***P*-value**	^**^r^2^
rs2493409	I	0.929	*NOTCH2*	1	120512104	T	C	0.084	0.385	0.139	0.009	rs10923931	0.269	0.929
rs13424212	I	0.979	*BCL11A*	2	207643224	G	A	0.943	−0.519	0.166	0.002	rs243088	0.826	0.979
rs726578	I	0.805	*RND3*	2	151644711	T	G	0.643	−0.217	0.084	0.024	rs7560163	0.125	0.805
rs116553151	G	NA	*ZPLD1*	3	102225887	G	A	0.978	0.631	0.242	0.019	rs2063640	0.842	0.98
rs143882978	I	0.958	*ADCY5*	3	123045588	C	T	0.948	−0.462	0.166	0.019	rs11708067	0.098	0.958
rs77144727	I	0.776	*WNT5A*	3	55313616	C	G	0.985	0.984	0.333	0.008	rs358806	0.588	0.776
rs76036930	I	0.915	*WFS1*	4	6300628	C	A	0.926	−0.411	0.153	0.021	rs1801214	0.912	0.915
rs35201724	I	0.756	*MAEA*	4	1310717	C	G	0.871	0.345	0.125	0.011	rs6815464	0.954	0.756
rs6856996	I	0.661	*TMEM155*	4	122671271	C	T	0.974	−0.797	0.335	0.035	rs7659604	0.504	0.661
rs148880354	I	0.948	*ANKRD55/MAP3K1*	5	55787227	G	A	0.976	0.745	0.235	0.002	rs459193	0.274	0.948
rs141867077	I	0.661	*ZFAND3*	6	38107234	C	T	0.988	−1.105	0.46	0.022	rs9470794	0.382	0.661
rs115694783	I	0.898	*ANK1*	8	41520264	G	A	0.962	0.546	0.198	0.014	rs516946	0.423	0.898
rs1328406	I	0.871	*TLE4*	9	81957798	C	T	0.661	0.231	0.081	0.014	rs13292136	0.154	0.871
rs34657422	G	NA	*PTPRD*	9	8902237	A	C	0.517	0.221	0.072	0.006	rs17584499	0.943	0.998
rs12414068	I	0.964	*TCERG1L*	10	132945800	A	G	0.908	−0.346	0.127	0.017	rs10741243	0.867	0.964
rs146170761	I	0.944	*CDC123*	10	12325422	C	T	0.96	−0.666	0.2	0.002	rs12779790	0.52	0.944
rs7115640	I	0.913	*TH/INS*	11	2194914	A	G	0.147	0.358	0.11	0.002	rs10770141	0.093	0.913
rs74728365	I	0.789	*KCNJ11*	11	17404846	A	C	0.95	0.46	0.184	0.026	rs5215	0.192	0.789
rs149672621	I	0.417	*MTNR1B*	11	92691694	A	G	0.984	−1.247	0.465	0.017	rs1387153	0.98	0.417
rs73419251	I	0.904	*KCNQ1*	11	17422709	A	G	0.956	−0.481	0.192	0.026	rs163184	0.515	0.904
rs76971568	I	0.921	*BARX2*	11	129474931	T	C	0.926	−0.451	0.148	0.004	rs7107217	0.122	0.921
rs149665582	I	0.943	*LOC1005 07205*	11	41920207	A	T	0.984	−0.792	0.303	0.017	rs9300039	0.652	0.943
rs115005036	I	0.765	*CCND2*	12	4373837	T	C	0.986	−1.214	0.402	0.003	rs11063069	0.754	0.765
rs75812308	I	0.932	*HMGA2*	12	66158505	A	G	0.72	−0.225	0.084	0.014	rs1531343	0.156	0.932
rs74102135	I	0.534	*TSPAN8*	12	71640010	C	G	0.986	−1.187	0.453	0.013	rs4760790	0.116	0.534
rs79164468	I	0.876	*C2CD4A/C2CD4B*	15	62405462	C	T	0.98	0.823	0.271	0.005	rs1436953	0.105	0.876
rs113762358	I	0.922	*PRC1*	15	91515135	G	A	0.889	0.341	0.117	0.007	rs8042680	0.359	0.922
rs56240666	I	0.971	*BCAR1*	16	75246825	C	G	0.381	−0.191	0.075	0.023	rs7202877	0.443	0.971
rs139888613	I	0.950	*MC4R*	18	57884481	G	A	0.973	0.823	0.24	0.002	rs12970134	0.752	0.95
rs148535989	I	0.675	*CILP2*	19	19391742	G	A	0.988	1.284	0.403	0.002	rs10401969	0.64	0.675

* *P-value adjusted for the number of tested SNPs in the LD region*.

** *r^2^ between reported SNP and SNP with best association signal*.

*NA, not applicable. Base-pair positions are in NCBI build 37 coordinates*.

Two of the 106 loci, *INS-IGF2* rs3842770, and *HLA-B* rs2244020, were reported by the only meta-analysis GWAS in an African ancestry population [the MEta-analysis of T2D in African Americans (MEDIA) Consortium, Ng et al., 2014]. In our sample of SSA, we found suggestive evidence of association for *INS-IGF2* rs3842770 (*p* = 0.067) and no significant association for *HLA-B* rs2244020 (merged into rs74995800, *p* = 0.878).

### Fine mapping

**Figure 1:**
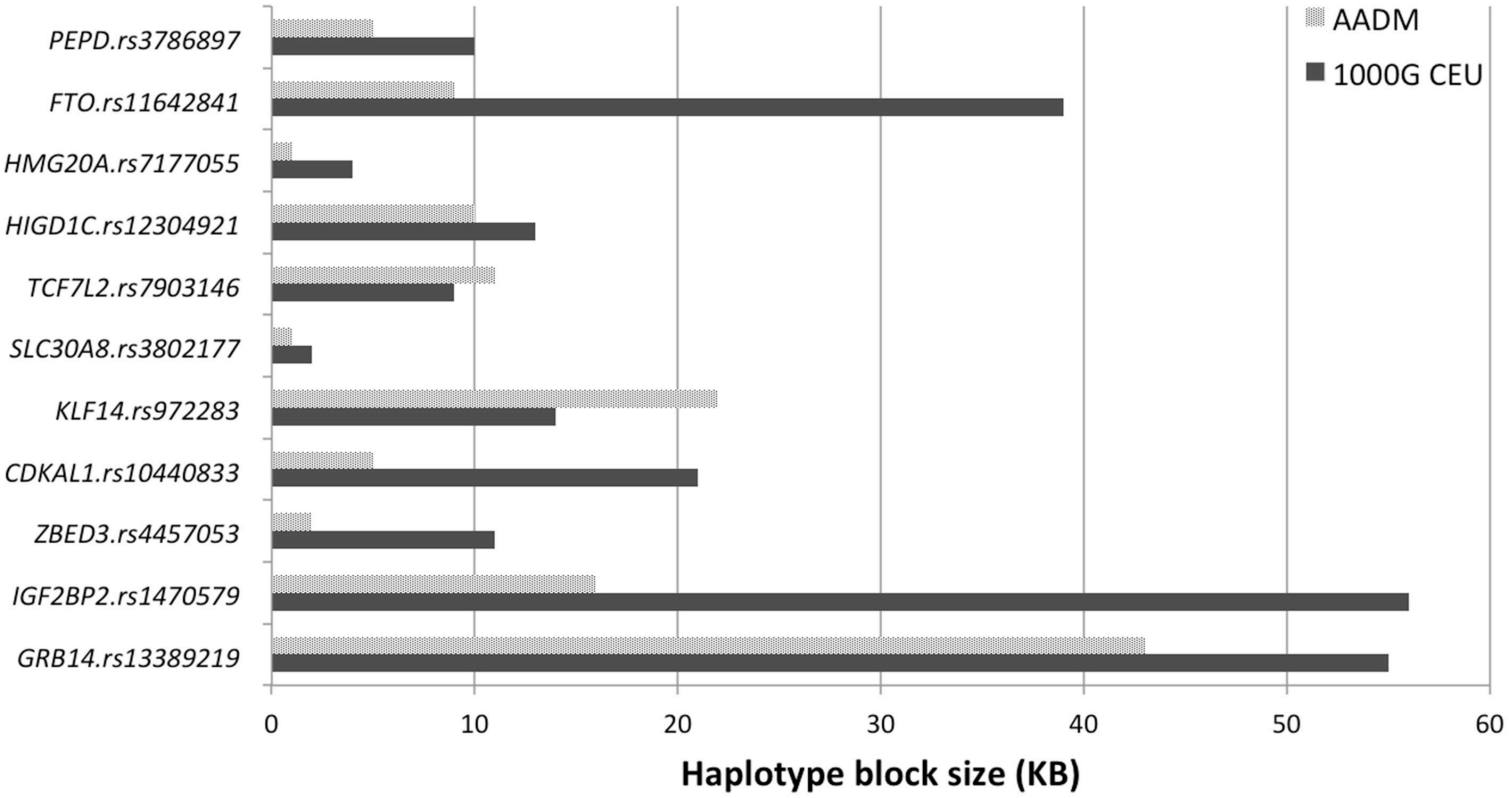
**Fine mapping of loci showing exact transferability in the AADM study**.

**Figure 2:**
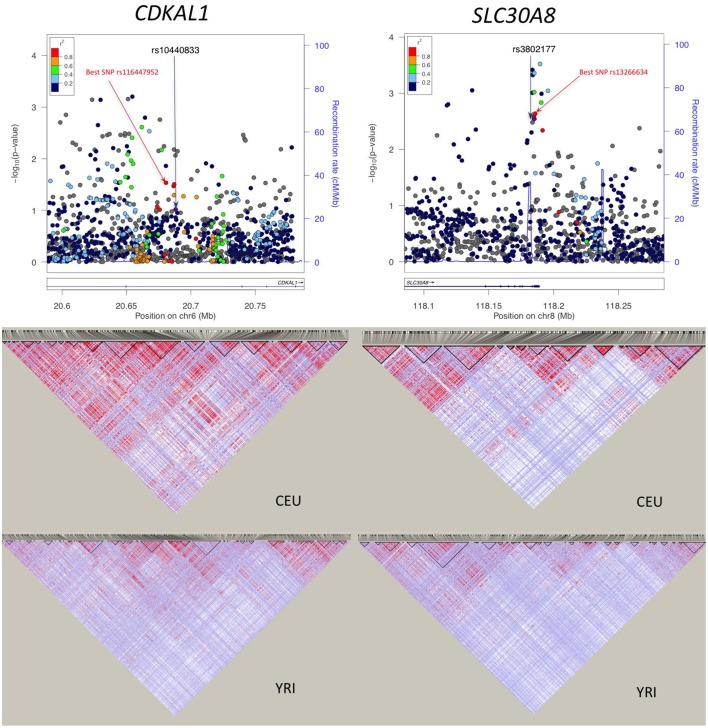
**Association plots and LD patterns at regions flanking *SLC30A8* and *CDKAL1***. The “best SNP” in the haplotype block is the SNP showing the smallest *p*-value that is in LD with the reported SNP.

For the 11 loci that showed exact transferability, we examined the LD structure around the index SNP to see if the locus could be fine-mapped. In 9 of the 11 loci, we found smaller haplotype block sizes around the lead SNPs in this study when compared to the original discovery population (Figure 1). Two examples, *SLC30A8* and *CDKAL1*, are shown in Figure 2. Notably, 9 of the 11 SNPs that showed exact transferability had another SNP in LD that showed stronger evidence of association (i.e., smaller *p*-values) than the reported index SNP (Supplementary Figure 5). The two exceptions in which the reported SNP also had the smallest *p*-value in the haplotype block were *TCF7L2* and *ZBED3*.

## Discussion

The field of T2D genetics has been remarkably successful in identifying risk loci using the GWAS approach, especially when multiple studies are combined in meta-analyses. Such studies of T2D (and other cardiovascular and metabolic diseases) remain rare in SSA. This study, evaluating for the first time a large number of reported T2D loci in individuals of African ancestry living on the continent, provides insight into the genetic architecture of T2D in SSA and promises to be a valuable resource for replication and meta-analysis as more GWAS are conducted in Africans. We focused on transferability of GWAS established T2D loci rather than discovery, given our limited sample size in the context of the known modest effect sizes of risk variants. We also conducted fine mapping studies, capitalizing on the lower LD and shorter haplotypes in populations of African ancestry. The low LD in African populations compared to European and Asian populations should make association studies in African ancestry populations a good way to fine map risk loci reported from large studies in these other populations. In addition, differences in diet, physical activity and other environmental factors could have an impact on association results, potentially improving the utility of African ancestry populations in genetic association studies.

We found evidence of transferability for 41 of 103 reported T2D loci tested in this study using both exact and local replication strategies. Our transferability rate for exact replication (11/103 or 10%) is somewhat lower than in earlier studies of African Americans. For example, Long et al. (2012) replicated 7 of 29 (24%) T2D associated SNPs while Ng et al. (2013) replicated 7 of 40 (18%) loci in their study of African Americans in the Candidate Gene Association Resources Plus Study. It is also lower than the 18% (19/104) transferability reported by a meta-analysis of African Americans (Ng et al., 2014). This is probably a reflection of sample size differences between the studies, since larger sample sizes have greater power to detect associations of a given effect size. Another factor that could account for these differences is that this study analyzed SSA living in Africa while the other studies were of African Americans: despite similar genetic ancestry, the environmental background is dramatically different, especially in terms of diet, physical activity, and obesity, all relevant for T2D risk.

*TCF7L2* rs7903146 showed the strongest association with T2D in this study. This locus is one of the most consistently replicated susceptibility loci for T2D in multiple populations. Notably, an African sample from the AADM study was instrumental to the refinement of the *TCF7L2* locus after its initial discovery (Grant et al., 2006; Helgason et al., 2007). Since then a number of candidate gene studies in Africans have confirmed its association with T2D in Ghana (Danquah et al., 2013), Cameroon (Guewo-Fokeng et al., 2015; Nanfa et al., 2015) and various North African groups (Bouhaha et al., 2010; Kifagi et al., 2011; Mtiraoui et al., 2012; Ben-Salem et al., 2014; Turki et al., 2014). Most of these studies have genotyped a few SNPs. A notable exception is an evaluation study of 37 GWAS-associated T2D loci in North African Arabs (Cauchi et al., 2012) which found nominal evidence for 13 of the loci reported in Europeans. In a wider context, the findings of this study are consistent with the expectation of observing differential effects when replicating tag SNPs found in European ancestry GWAS in non-European ancestry populations. This observation is most pronounced in African ancestry individuals with differential effects diluted toward the null (Carlson et al., 2013).

An expectation of association studies of African ancestry populations is that it would be possible to fine map or refine disease-associated loci because of lower LD and smaller haplotypes. The first demonstration of this principle for T2D was for the *TCF7L2* locus (Helgason et al., 2007). Several other studies have demonstrated the same phenomenon for T2D (Ng et al., 2013), as well as for glucose-related traits (Ramos et al., 2011), uric acid (Charles et al., 2011), bilirubin levels (Chen et al., 2012), and serum lipids (Adeyemo et al., 2012) in African Americans. In the present study, the majority of loci that showed transferability were fine-mapped with neighboring SNPs showing stronger association with T2D than the reported index SNP. Together, these findings provide compelling evidence that the reduced and different LD patterns present in African populations can facilitate trans-ethnic fine mapping of disease loci. It is therefore expected that the number of loci that can be fine mapped will increase as more studies are done in African ancestry populations.

Other than for replication and fine mapping, discovery studies in populations of different ancestries are needed as they have the potential to find novel susceptibility loci which could be population-specific or cosmopolitan yet more easily discovered in a specific population (McCarthy, 2008). Notable examples include the discovery of the T2D associated genes *KCNQ1* in East Asians (Yasuda et al., 2008; Unoki et al., 2008), *SGCG* in Punjabi Sikhs (Saxena et al., 2013) and of *SLC6A11* in Mexicans (SIGMA Type 2 Diabetes Consortium et al., 2014). Given the genetic and environmental diversity represented on the African continent, doing such studies in African populations has the potential to discover novel loci and enrich our knowledge of the genetics of T2D on the continent. In addition, similar to the European and Asian experiences, it is expected that more shared T2D loci across global populations will be discovered as additional studies are conducted in Africans and larger sample sizes become available for meta-analysis.

A potential limitation in the present study is the sample size. Larger samples have the potential to identify and replicate more T2D risk loci, especially those with smaller effect sizes or with lower allele frequencies. Nonetheless, the study provides a resource for future studies of T2D in Africans for purposes of replication and meta-analysis.

In conclusion, this first large scale replication and fine mapping analysis of reported T2D-associated risk loci in Africans successfully demonstrated evidence of transferability and trans-ethnic fine mapping of several loci reported in European and Asian ancestry populations. Notably, 41 reported GWAS loci for T2D were found to be associated with disease risk in this study. These findings indicate that the genetic architecture of T2D in SSA is characterized by several risk loci shared with non-African ancestral populations and that data from African populations may facilitate fine mapping of risk loci.

## Author contributions

CR, AA, GD, FC designed the study; OF, TJ, BE, KA, JA, WB, CA, AA2, JA, DC, CA, GO, JO did participant recruitment, phenotyping and field laboratory assays; AD, HH, AE, SC did molecular laboratory assays and genotyping; AA, FT, AB, JZ, GC, DS did data management and statistical analysis; AA, FT, AB drafted the manuscript; CR, DS, FC edited the manuscript; all authors reviewed and approved the manuscript.

## Conflict of interest statement

The authors declare that the research was conducted in the absence of any commercial or financial relationships that could be construed as a potential conflict of interest.

## References

[B1] Adeyemo A. Bentley A. R. Meilleur K. G. Doumatey A. P. Chen G. Zhou J.. (2012). Transferability and fine mapping of genome-wide associated loci for lipids in African Americans. BMC Med. Genet. 13:88. 10.1186/1471-2350-13-88 22994408 PMC3573912

[B2] Ben-Salem A. Ajina M. Suissi M. Daher H. S. Almawi W. Y. Mahjoub T. (2014). Polymorphisms of transcription factor-7-like 2 (TCF7L2) gene in Tunisian women with polycystic ovary syndrome (PCOS). Gene 533, 554–557. 10.1016/j.gene.2013.09.104 24157263

[B3] Bouhaha R. Choquet H. Meyre D. Abid Kamoun H. Ennafaa H. Baroudi T.. (2010). TCF7L2 is associated with type 2 diabetes in nonobese individuals from Tunisia. Pathol. Biol. 58, 426–429. 10.1016/j.patbio.2009.01.003 19286335

[B4] Bretherton C. S. Widmann M. Dymnikov V. P. Wallace J. M. Blade I. (1999). The effective number of spatial degrees of freedom of a time-varying field. J. Clim. 12, 1990–2009.

[B5] Carlson C. S. Matise T. C. North K. E. Haiman C. A. Fesinmeyer M. D. Buyske S.. (2013). Generalization and dilution of association results from European GWAS in populations of non-European ancestry: the PAGE study. PLoS Biol. 11:e1001661. 10.1371/journal.pbio.1001661 24068893 PMC3775722

[B6] Cauchi S. Ezzidi I. El Achhab Y. Mtiraoui N. Chaieb L. Salah D.. (2012). European genetic variants associated with type 2 diabetes in North African Arabs. Diabetes Metab. 38, 316–323. 10.1016/j.diabet.2012.02.003 22463974

[B7] Charles B. A. Shriner D. Doumatey A. Chen G. Zhou J. Huang H.. (2011). A genome-wide association study of serum uric acid in African Americans. BMC Med. Genomics 4:17. 10.1186/1755-8794-4-17 21294900 PMC3045279

[B8] Chen G. Ramos E. Adeyemo A. Shriner D. Zhou J. Doumatey A. P.. (2012). UGT1A1 is a major locus influencing bilirubin levels in African Americans. Eur. J. Hum. Genet. 20, 463–468. 10.1038/ejhg.2011.206 22085899 PMC3306855

[B9] Danquah I. Othmer T. Frank L. K. Bedu-Addo G. Schulze M. B. Mockenhaupt F. P. (2013). The TCF7L2 rs7903146 (T) allele is associated with type 2 diabetes in urban Ghana: a hospital-based case-control study. BMC Med. Genet. 14:96. 10.1186/1471-2350-14-96 24059590 PMC3848778

[B10] Gabriel S. B. Schaffner S. F. Nguyen H. Moore J. M. Roy J. Blumenstiel B.. (2002). The structure of haplotype blocks in the human genome. Science 296, 2225–2229. 10.1126/science.1069424 12029063

[B11] Grant S. F. Thorleifsson G. Reynisdottir I. Benediktsson R. Manolescu A. Sainz J.. (2006). Variant of transcription factor 7-like 2 (TCF7L2) gene confers risk of type 2 diabetes. Nat. Genet. 38, 320–323. 10.1038/ng1732 16415884

[B12] Guewo-Fokeng M. Sobngwi E. Atogho-Tiedeu B. Donfack O. S. Noubiap J. J. Ngwa E. N.. (2015). Contribution of the TCF7L2 rs7903146 (C/T) gene polymorphism to the susceptibility to type 2 diabetes mellitus in Cameroon. J. Diabetes Metab. Disord. 14, 26. 10.1186/s40200-015-0148-z 25897419 PMC4403887

[B13] Helgason A. Palsson S. Thorleifsson G. Grant S. F. Emilsson V. Gunnarsdottir S.. (2007). Refining the impact of TCF7L2 gene variants on type 2 diabetes and adaptive evolution. Nat. Genet. 39, 218–225. 10.1038/ng1960 17206141

[B14] Kifagi C. Makni K. Boudawara M. Mnif F. Hamza N. Abid M.. (2011). Association of genetic variations in TCF7L2, SLC30A8, HHEX, LOC387761, and EXT2 with Type 2 diabetes mellitus in Tunisia. Genet. Test. Mol. Biomarkers 15, 399–405. 10.1089/gtmb.2010.0199 21510814

[B15] IDF (2013). IDF Diabetes Atlas [Online]. Brussels: International Diabetes Federation Available online at: http://www.idf.org/diabetesatlas

[B16] Li Y. Willer C. J. Ding J. Scheet P. Abecasis G. R. (2010). MaCH: using sequence and genotype data to estimate haplotypes and unobserved genotypes. Genet. Epidemiol. 34, 816–834. 10.1002/gepi.20533 21058334 PMC3175618

[B17] Liu E. Y. Li M. Wang W. Li Y. (2013). MaCH-admix: genotype imputation for admixed populations. Genet. Epidemiol. 37, 25–37. 10.1002/gepi.21690 23074066 PMC3524415

[B18] Long J. Edwards T. Signorello L. B. Cai Q. Zheng W. Shu X. O.. (2012). Evaluation of genome-wide association study-identified type 2 diabetes loci in African Americans. Am. J. Epidemiol. 176, 995–1001. 10.1093/aje/kws176 23144361 PMC3571243

[B19] McCarthy M. I. (2008). Casting a wider net for diabetes susceptibility genes. Nat. Genet. 40, 1039–1040. 10.1038/ng0908-1039 19165915

[B20] Morris A. P. Voight B. F. Teslovich T. M. Ferreira T. Segrè A. V. Steinthorsdottir V.. (2012). Large-scale association analysis provides insights into the genetic architecture and pathophysiology of type 2 diabetes. Nat. Genet. 44, 981–990. 10.1038/ng.2383 22885922 PMC3442244

[B21] Mtiraoui N. Turki A. Nemr R. Echtay A. Izzidi I. Al-Zaben G. S.. (2012). Contribution of common variants of ENPP1, IGF2BP2, KCNJ11, MLXIPL, PPARgamma, SLC30A8 and TCF7L2 to the risk of type 2 diabetes in Lebanese and Tunisian Arabs. Diabetes Metab. 38, 444–449. 10.1016/j.diabet.2012.05.002 22749234

[B22] Nanfa D. Sobngwi E. Atogho-Tiedeu B. Noubiap J. J. Donfack O. S. Mofo E. P.. (2015). Association between the TCF7L2 rs12255372 (G/T) gene polymorphism and type 2 diabetes mellitus in a Cameroonian population: a pilot study. Clin. Transl. Med. 4, 17. 10.1186/s40169-015-0058-1 25995831 PMC4434239

[B23] Ng M. C. Saxena R. Li J. Palmer N. D. Dimitrov L. Xu J.. (2013). Transferability and fine mapping of type 2 diabetes loci in African Americans: the Candidate Gene Association Resource Plus Study. Diabetes 62, 965–976. 10.2337/db12-0266 23193183 PMC3581206

[B24] Ng M. C. Shriner D. Chen B. H. Li J. Chen W. M. Guo X.. (2014). Meta-analysis of genome-wide association studies in african americans provides insights into the genetic architecture of type 2 diabetes. PLoS Genet. 10:e1004517. 10.1371/journal.pgen.1004517 25102180 PMC4125087

[B25] Ramos E. Chen G. Shriner D. Doumatey A. Gerry N. P. Herbert A.. (2011). Replication of genome-wide association studies (GWAS) loci for fasting plasma glucose in African-Americans. Diabetologia 54, 783–788. 10.1007/s00125-010-2002-7 21188353 PMC3052446

[B26] Rotimi C. N. Chen G. Adeyemo A. A. Furbert-Harris P. Parish-Gause D. Zhou J.. (2004). A genome-wide search for type 2 diabetes susceptibility genes in West Africans: the Africa America Diabetes Mellitus (AADM) Study. Diabetes 53, 838–841. 10.2337/diabetes.53.3.838 14988271

[B27] Rotimi C. N. Dunston G. M. Berg K. Akinsete O. Amoah A. Owusu S.. (2001). In search of susceptibility genes for type 2 diabetes in West Africa: the design and results of the first phase of the AADM study. Ann. Epidemiol. 11, 51–58. 10.1016/S1047-2797(00)00180-0 11164120

[B28] Saxena R. Saleheen D. Been L. F. Garavito M. L. Braun T. Bjonnes A.. (2013). Genome-wide association study identifies a novel locus contributing to type 2 diabetes susceptibility in Sikhs of Punjabi origin from India. Diabetes 62, 1746–1755. 10.2337/db12-1077 23300278 PMC3636649

[B29] Sigma Type 2 Diabetes Consortium Williams, A. L. Jacobs S. B. Moreno-Macias H. Huerta-Chagoya A. Churchhouse C.. (2014). Sequence variants in *SLC16A11* are a common risk factor for type 2 diabetes in Mexico. Nature 506, 97–101. 10.1038/nature12828 24390345 PMC4127086

[B30] Turki A. Al-Zaben G. S. Khirallah M. Marmouch H. Mahjoub T. Almawi W. Y. (2014). Gender-dependent associations of CDKN2A/2B, KCNJ11, POLI, SLC30A8, and TCF7L2 variants with type 2 diabetes in (North African) Tunisian Arabs. Diabetes Res. Clin. Pract. 103, e40–e43. 10.1016/j.diabres.2013.12.040 24485399

[B31] Unoki H. Takahashi A. Kawaguchi T. Hara K. Horikoshi M. Andersen G.. (2008). SNPs in *KCNQ1* are associated with susceptibility to type 2 diabetes in East Asian and European populations. Nat. Genet. 40, 1098–1102. 10.1038/ng.208 18711366

[B32] Wild S. Roglic G. Green A. Sicree R. King H. (2004). Global prevalence of diabetes: estimates for the year 2000 and projections for 2030. Diabetes Care 27, 1047–1053. 10.2337/diacare.27.5.1047 15111519

[B33] Yasuda K. Miyake K. Horikawa Y. Hara K. Osawa H. Furuta H.. (2008). Variants in *KCNQ1* are associated with susceptibility to type 2 diabetes mellitus. Nat. Genet. 40, 1092–1097. 10.1038/ng.207 18711367

