# Supplementary material for: Evaluation of Genome Wide Association Study Associated Type 2 Diabetes Susceptibility Loci in Sub Saharan Africans
Source: Front Genet. 2015 Nov 24;6:335. doi: 10.3389/fgene.2015.00335 (PMC4656823; doi:10.3389/fgene.2015.00335)
Supplement: Supplementary file 1 [file DataSheet1.PDF]

Supplementary material to:

Evaluation of Genome Wide Association Study Associated Type 2 Diabetes Susceptibility Loci in Sub Saharan Africans

Adebowale A. Adeyemo,[1] Fasil Tekola-Ayele,[1] Ayo P. Doumatey,[1] Amy R. Bentley,[1] Guanjie Chen,[1] Hanxia Huang,[1] Jie Zhou,[1] Daniel Shriner,[1] Olufemi Fasanmade,[2] Godfrey Okafor,[3] Benjamin Eghan Jr,[4] Kofi Agyenim-Boateng,[4] Jokotade Adeleye,[5] Williams Balogun,[5] Abdel Elkahloun,[6] Settara Chandrasekharappa,[6] Samuel Owusu,[7] Albert Amoah,[7] Joseph Acheampong,[4] Thomas Johnson,[2] Johnnie Oli,[3] Clement Adebamowo,[8] Francis Collins,[9] Georgia Dunston,[10] Charles N. Rotimi[1*]

[1]Center for Research on Genomics and Global Health, National Human Genome Research Institute, National Institutes of Health, Bethesda, MD, USA

[2]University of Lagos, Lagos, Nigeria

[3]University of Nigeria Teaching Hospital, Enugu, Nigeria

[4]University of Science and Technology, Kumasi, Ghana

[5]University of Ibadan, Ibadan, Nigeria

[6]National Human Genome Research Institute, National Institutes of Health, Bethesda, MD, USA

[7]University of Ghana Medical School, Accra, Ghana

[8]Institute of Human Virology, School of Medicine, University of Maryland, Baltimore, MD, USA

[9]National Institutes of Health, Bethesda, MD, USA

[10]National Human Genome Center at Howard University, Washington, DC, USA

***Corresponding author:** Charles Rotimi, PhD, Center for Research on Genomics and Global Health, National Human Genome Research Institute, Building 12A, Room 4047, 12 South Drive, MSC 5635, Bethesda, MD 20892-5635, USA.

E-mail: rotimic@mail.nih.gov

Supplementary Material Table of Contents

Supplementary Table 1: Individuals and SNPs passing quality control (QC)

Supplementary Table 2: List of type 2 diabetes associated SNPs tested for transferability and fine mapping

Supplementary Table 3: Top scoring loci for association with type 2 diabetes in AADM under an additive genetic model

Supplementary Table 4: Top scoring loci for association with type 2 diabetes in AADM under an additive genetic model with no adjustment for body mass index (BMI)

Supplementary Table 5: Association statistics of 33 loci based on genotype data versus imputed dosages

Supplementary Figure 1: Allele frequency spectrum of SNPs genotyped on Affymetrix Axiom PanAFR® array

Supplementary Figure 2: PCA plots of the 1775 subjects

Supplementary Figure 3: QQ plot of association with type 2 diabetes in AADM

Supplementary Figure 4: Power of the study for replication at a one-sided α of 0.05

Supplementary Figure 5: Plots of regional p-values and LD patterns at loci showing exact transferability in AADM

Supplementary Table 1: Individuals and SNPs passing quality control (QC)

	Individuals Excluded	**Remaining Individuals**	SNPs Excluded	**Remaining SNPs**
**Initial Dataset**		**1822**		**2,217,748**
Sample Exclusions				
- Duplicate sample	1	1821		
- Sex discordant samples	13	1808		
- Related individuals	33	1775		
Marker Exclusions				
- Missing for more than 5% of individuals			94,438	2,123,310
- HWE $p<10^{-6}$			20,472	2,102,838
- MAF<0.01			45,759	2,057,079
- Non-autosomal SNPs			58,171	1,998,908
**Reference Dataset**		**1775**		1,998,908

Supplementary Table 2: List of type 2 diabetes associated SNPs tested for transferability and fine mapping

SNP	CHR	Position (hg19)	GENE
rs7542900	1	95070041	ABCD3, F3, SLC44A3
rs10923931	1	120517959	NOTCH2
rs17045328	1	207652176	CR2
rs12027542	1	233340154	PCNXL2
rs4659485	1	237145918	MTR, RYR2
rs11677370	2	3841420	DCDC2C
rs7578597	2	43732823	THADA
rs243088	2	60568745	BCL11A
rs6712932	2	105837598	GPR45
rs7560163	2	151637936	RND3
rs7593730	2	161171454	RBMS1
rs13389219	2	165528876	GRB14
rs7578326	2	227020653	IRS1
rs10497721	2	192914362	TMEFF2
rs1801282	3	12393125	PPARG
rs6784615	3	52506426	NISCH
rs358806	3	55313400	WNT5A
rs831571	3	64048297	PSMD6
rs4607103	3	64711904	ADAMTS9
rs2063640	3	102203045	ZPLD1
rs11708067	3	123065778	ADCY5
rs3773506	3	142431000	PLS1
rs7630877	3	179661318	PEX5L
rs1470579	3	185529080	IGF2BP2
rs16861329	3	186666461	ST6GAL1
rs6815464	4	1309901	MAEA
rs1801214	4	6303022	WFS1
rs7659604	4	122665514	TMEM155
rs3792615	4	164532801	MARCH1
rs459193	5	55806751	ANKRD55, MAP3K1
rs4457053	5	76424949	ZBED3
rs12518099	5	89546109	CETN3
rs10440833	6	20688121	CDKAL1
rs2244020	6	31347451	HLA-B
rs9470794	6	38106844	ZFAND3
rs1535500	6	39284050	KCNK16
rs9472138	6	43811762	VEGFA
rs1048886	6	71289189	C6orf57
rs2021966	6	132150439	ENPP1
rs642858	6	140273647	LOC100132735
rs17168486	7	14898282	DGKB
rs849134	7	28196222	JAZF1
rs7636	7	100490077	ACHE
rs6467136	7	127164958	GCC1, PAX4
rs1882095	7	129397644	NRF1
rs972283	7	130466854	KLF14
rs516946	8	41519248	ANK1
rs896854	8	95960511	TP53INP1
rs3802177	8	118185025	SLC30A8
rs7041847	9	4287466	GLIS3
rs17584499	9	8879118	PTPRD
rs649891	9	10430602	PTPRD
rs564398	9	22029547	CDKN2B-AS
rs10965250	9	22133284	CDKN2B-AS
rs13292136	9	81952128	TLE4

rs2796441	9	84308948	TLE1
rs4457406	9	115893544	SLC31A2
rs4740283	9	134448296	RAPGEF1
rs12779790	10	12328010	CDC123
rs1802295	10	70931474	VPS26A
rs12571751	10	80942631	ZMIZ1
rs1111875	10	94462882	HHEX
rs7903146	10	114758349	TCF7L2
rs10741243	10	132947962	TCERG1L
rs10770141	11	2193840	TH, INS
rs231362	11	2691471	KCNQ1
rs163184	11	2847069	KCNQ1
rs2722769	11	11228374	GALNTL4
rs5215	11	17408630	KCNJ11
rs3842770	11	2178670	INS-IGF2
rs9300039	11	41915366	LOC100507205
rs1552224	11	72433098	ARAP1
rs1387153	11	92673828	MTNR1B
rs7107217	11	129473690	BARX2
rs11063069	12	4374373	CCND2
rs718314	12	26453283	ITPR2, SSPN
rs10842994	12	27965150	KLHDC5
rs12304921	12	51357542	HIGD1C
rs1153188	12	55098996	DCD
rs1531343	12	66174894	HMGA2
rs1495377	12	71577101	TSPAN8
rs4760790	12	71634794	TSPAN8
rs7957197	12	121460686	HNF1A
rs1359790	13	80717156	SPRY2
rs730570	14	101142890	DLK1
rs7172432	15	62396389	C2CD4A, C2CD4B
rs1436953	15	62414014	C2CD4A, C2CD4B
rs7177055	15	77832762	HMG20A
rs11634397	15	80432222	ZFAND6
rs2028299	15	90374257	AP3S2
rs8042680	15	91521337	PRC1
rs11642841	16	53845487	FTO
rs7202877	16	75247245	BCAR1
rs391300	17	2216258	SRR
rs1042522	17	7579472	TP53
rs4925115	17	17721457	SREBF1
rs4430796	17	36098040	HNF1B
rs10460009	18	2948029	LPIN2
rs12970134	18	57884750	MC4R
rs12454712	18	60845884	BCL2
rs10401969	19	19407718	CILP2
rs3786897	19	33893008	PEPD
rs472265	19	39580737	PAPL
rs8108269	19	46158513	GIPR
rs4812829	20	42989267	HNF4A
rs2833610	21	33385186	HUNK

Supplementary Table 3: Top scoring loci for association with type 2 diabetes in AADM under an additive genetic model*

SNP	CHR	BP	A1	A2	A1 Freq	R2	Effect1	OR	SE	Chi2	P
rs7903146	10	114758349	C	T	.6718	.8916	-0.465	0.628	0.084	31.9198	1.607e-08
rs4608622	22	33268413	C	T	.9327	.9865	0.793	2.210	0.149	29.5439	5.466e-08
rs5754319	22	33270348	C	A	.9329	.9915	0.790	2.203	0.149	29.4114	5.853e-08
rs5754322	22	33272699	T	C	.9328	.9736	0.795	2.214	0.150	29.3029	6.19e-08
rs17778906	22	33274175	C	G	.9656	.8928	1.156	3.176	0.225	29.1518	6.693e-08
rs11704745	22	33268517	T	A	.9668	.9618	1.118	3.058	0.220	28.4375	9.677e-08
rs5754321	22	33270461	T	C	.9324	.9941	0.751	2.119	0.147	27.2665	1.773e-07
rs186237038	9	115146604	A	G	.9975	.4412	-24.702	0.000	7.171	27.0864	1.946e-07
rs76561119	17	50422383	T	C	.9881	.8299	-2.757	0.064	0.778	26.9635	2.073e-07
rs142306327	1	19593447	G	A	.9990	.5340	-92.170	0.000	30.936	26.3926	2.786e-07
rs111694730	22	47022116	A	G	.9958	.3222	-8.760	0.000	2.832	26.2303	3.03e-07
rs148554359	17	50415273	G	C	.9866	.7977	-2.467	0.085	0.658	26.0237	3.373e-07
rs115221558	22	33275768	C	T	.9886	.8980	1.964	7.128	0.448	25.8468	3.696e-07
rs4506565	10	114756041	A	T	.5051	.9070	-0.380	0.684	0.076	25.7791	3.828e-07
rs318457	6	2938122	T	G	.8320	.8999	0.494	1.638	0.099	25.5203	4.378e-07
rs73987697	17	50424744	A	G	.9897	.7528	-2.977	0.051	0.864	25.3586	4.76e-07
rs7901695	10	114754088	T	C	.5055	.9112	-0.376	0.687	0.075	25.3234	4.848e-07
rs73987698	17	50424805	T	C	.9896	.7521	-2.963	0.052	0.860	25.2508	5.034e-07

*Model was for age, sex, BMI and first 3 PCs

Supplementary Table 4: Top scoring loci for association with type 2 diabetes in AADM under an additive genetic model with no adjustment for body mass index*

SNP	CHR	BP	A1	A2	A1 Freq	R2	Effect1	OR	SE	Chi2	P
rs7903146	10	114758349	C	T	.6718	.8916	-0.446	0.640	0.083	29.7121	5.012e-08
rs4608622	22	33268413	C	T	.9327	.9865	0.785	2.193	0.149	29.1334	6.756e-08
rs5754319	22	33270348	C	A	.9329	.9915	0.782	2.187	0.149	29.0128	7.19e-08
rs5754322	22	33272699	T	C	.9328	.9736	0.787	2.197	0.150	28.8971	7.633e-08
rs17778906	22	33274175	C	G	.9656	.8928	1.143	3.135	0.224	28.7071	8.419e-08
rs76561119	17	50422383	T	C	.9881	.8299	-2.805	0.061	0.775	28.1479	1.124e-07
rs11704745	22	33268517	T	A	.9668	.9618	1.107	3.025	0.219	28.0623	1.175e-07
rs148554359	17	50415273	G	C	.9866	.7977	-2.514	0.081	0.656	27.1823	1.851e-07
rs5754321	22	33270461	T	C	.9324	.9941	0.744	2.104	0.147	26.8850	2.159e-07
rs111694730	22	47022116	A	G	.9958	.3222	-8.821	0.000	2.827	26.6021	2.5e-07
rs73987697	17	50424744	A	G	.9897	.7528	-3.037	0.048	0.864	26.5445	2.575e-07
rs142306327	1	19593447	G	A	.9990	.5340	-92.369	0.000	30.922	26.5179	2.611e-07
rs73987698	17	50424805	T	C	.9896	.7521	-3.022	0.049	0.859	26.4217	2.744e-07
rs186237038	9	115146604	A	G	.9975	.4412	-24.277	0.000	7.122	26.1950	3.086e-07
rs73987692	17	50407757	T	C	.9883	.8006	-2.611	0.073	0.701	26.1571	3.147e-07
rs73987693	17	50409535	C	T	.9856	.8145	-2.244	0.106	0.568	25.9795	3.451e-07
rs73987670	17	50394692	A	G	.9886	.8077	-2.620	0.073	0.708	25.8126	3.762e-07

*Model was for age, sex, BMI and first 3 PCs

Supplementary Table 5: Association statistics of 33 SNPs based on genotype data versus imputed dosages

SNP	CHR	BP	A1	A2	Genotype association		Imputed dosage association		Imputation R^2 metric
					OR	P	OR	P	
rs7542900	1	95070041	T	C	0.9478	0.4485	0.968	0.6427	0.9983
rs7578326	2	227020653	A	G	0.9236	0.2772	0.96	0.5711	0.9835
rs358806	3	55313400	A	C	1.062	0.6104	1.067	0.5871	0.9833
rs831571	3	64048297	T	C	0.9974	0.9772	1.021	0.826	0.9958
rs4607103	3	64711904	C	T	1.062	0.4509	1.111	0.1706	0.9915
rs2063640	3	102203045	C	A	0.9927	0.9703	0.962	0.841	0.9806
rs11708067	3	123065778	A	G	1.176	0.1286	1.192	0.09906	0.9987
rs1470579	3	185529080	A	C	0.6745	0.0001111	0.706	0.0005158	0.9938
rs16861329	3	186666461	C	T	1.028	0.8641	1.022	0.8958	0.9974
rs7659604	4	122665514	C	T	1.056	0.4979	1.054	0.5088	0.999
rs4457053	5	76424949	G	A	1.344	0.003773	1.32	0.005895	0.987
rs12518099	5	89546109	A	G	1.004	0.9678	1.003	0.9739	0.9925
rs1048886	6	71289189	A	G	0.9837	0.8355	0.992	0.9158	0.9936
rs2021966	6	132150439	A	G	0.898	0.1828	0.904	0.2072	0.9896
rs17168486	7	14898282	C	T	0.8127	0.1189	0.795	0.08429	0.9988
rs7636	7	100490077	G	A	0.9178	0.2756	0.915	0.2526	0.9939
rs1882095	7	129397644	T	C	0.9094	0.1783	0.92	0.2373	0.9976
rs972283	7	130466854	A	G	0.7565	0.03455	0.76	0.03719	0.9951
rs516946	8	41519248	T	C	1.028	0.751	1.071	0.4179	0.986
rs17584499	9	8879118	C	T	0.9509	0.8217	0.983	0.9409	0.9692
rs2796441	9	84308948	G	A	1.209	0.1093	1.24	0.06598	0.99
rs7107217	11	129473690	A	C	1.093	0.2157	1.115	0.1259	0.9956
rs11063069	12	4374373	A	G	1.001	0.992	1.028	0.7596	0.9753
rs718314	12	26453283	A	G	0.9885	0.9031	0.978	0.8115	0.9983
rs12304921	12	51357542	A	G	0.7974	0.01872	0.782	0.01042	0.9981
rs1359790	13	80717156	G	A	0.9129	0.5205	0.938	0.6548	0.9872
rs7177055	15	77832762	G	A	0.8645	0.0744	0.839	0.0288	0.9953
rs8042680	15	91521337	C	A	1.279	0.379	1.3	0.3573	0.998
rs7202877	16	75247245	T	G	1.08	0.4388	1.078	0.4467	0.9988
rs12454712	18	60845884	T	C	1.127	0.2009	1.116	0.2508	0.9284
rs472265	19	39580737	A	G	1.075	0.3676	1.119	0.1554	0.9895
rs8108269	19	46158513	T	G	1.074	0.3253	1.07	0.3472	0.9925
rs2833610	21	33385186	A	G	1.068	0.3946	1.062	0.4294	0.994

Supplementary Figure 1: Allele frequency spectrum of SNPs genotyped on Affymetrix Axiom PanAFR® array

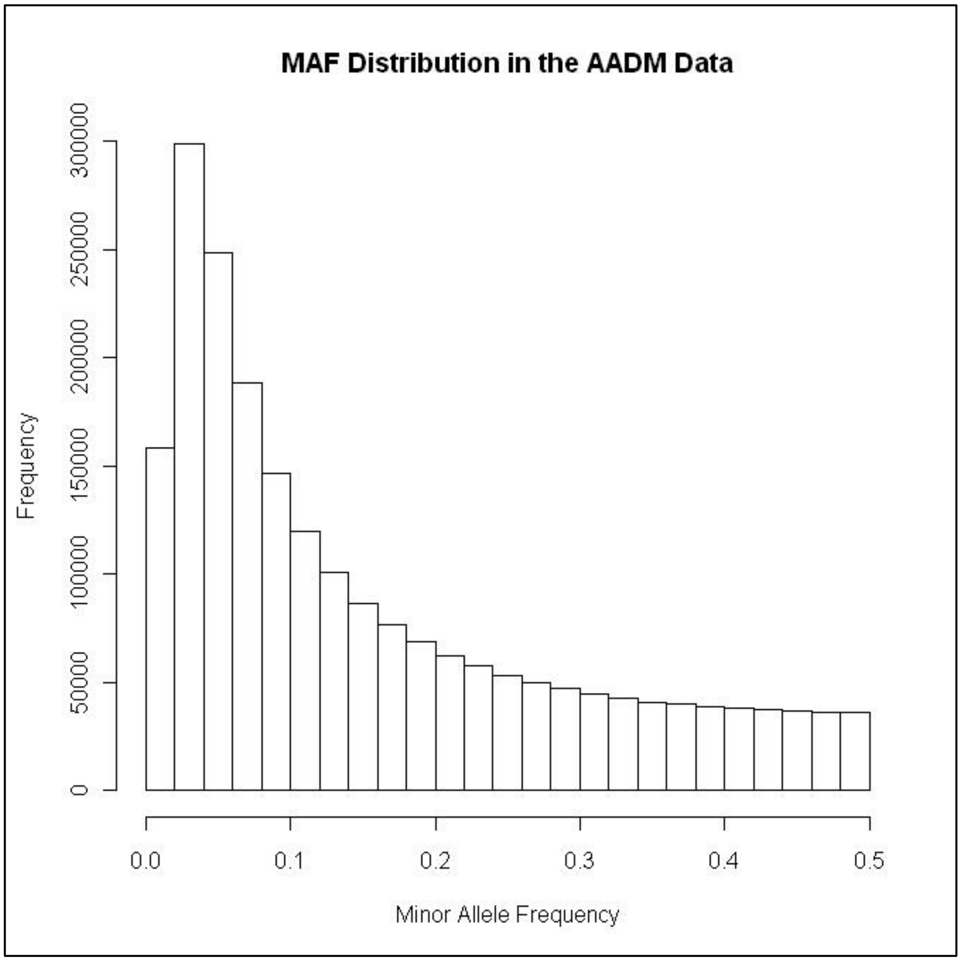

Supplementary Figure 2: PCA plots of the 1775 subjects

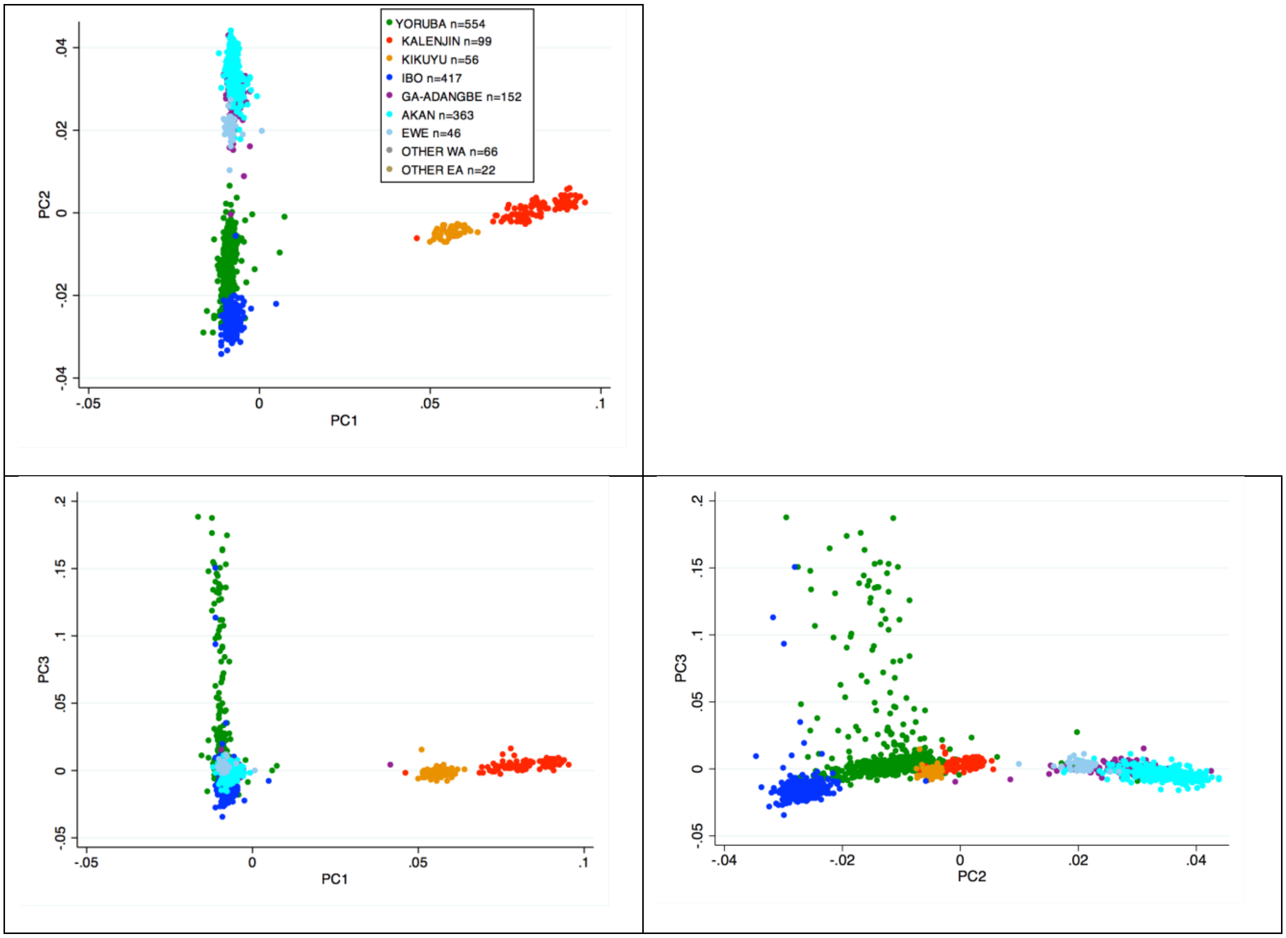

Supplementary Figure 3: QQ plot of association statistics for type 2 diabetes in AADM

(a) Model adjusted for age, sex, BMI and first 3 PCs

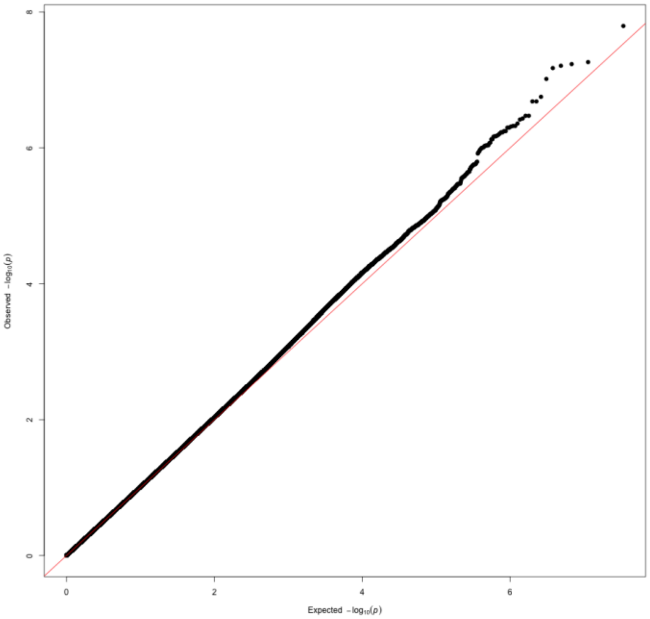

(b) Model adjusted for age, sex and first 3 PCs (without BMI)

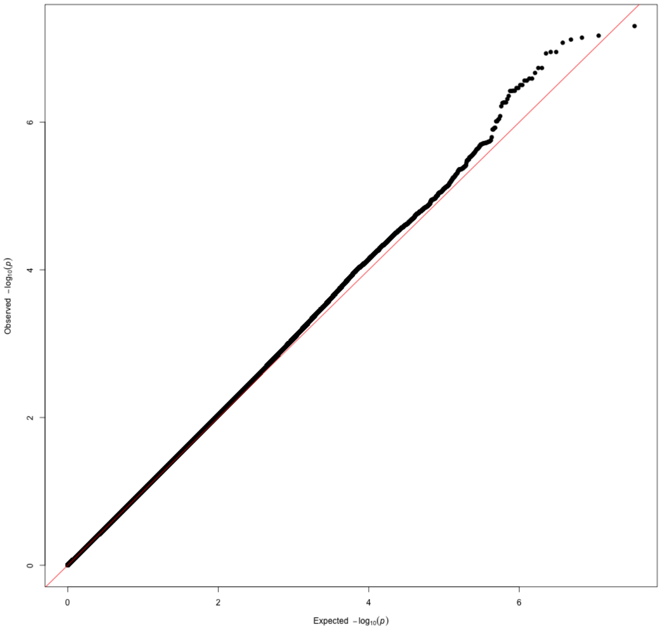

Supplementary Figure 4: Power of the study for replication at a one-sided α of 0.05 at various minor allele frequencies

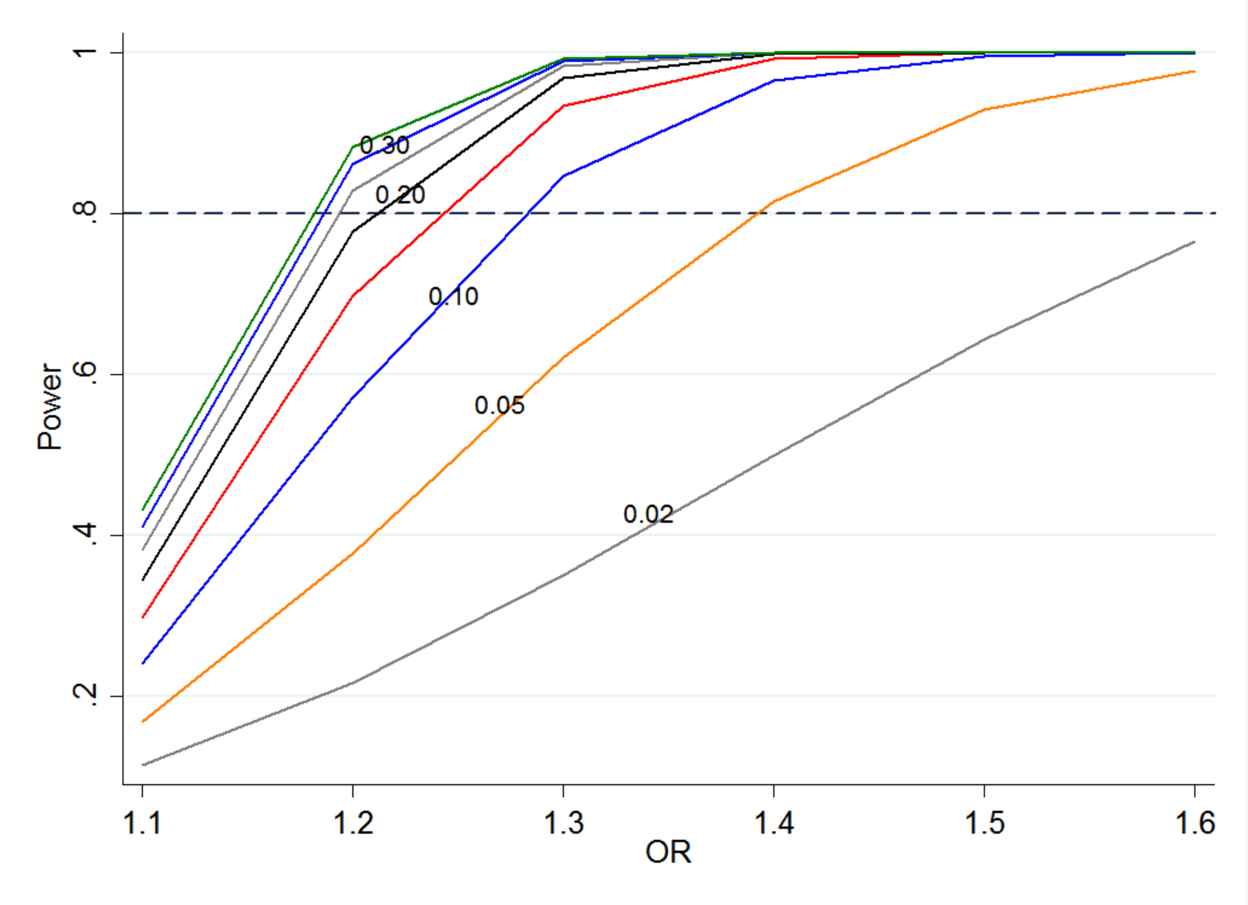

Supplementary Figure 5: Plots of regional p-values and LD patterns at loci showing exact transferability in AADM

Note: SNPs are color coded relative to the degree of LD with the index SNP (see legend). SNPs with missing LD information are shown in grey. Index SNP=reported SNP ; best SNP= the most significant SNP in LD with the reported SNP

GRB14

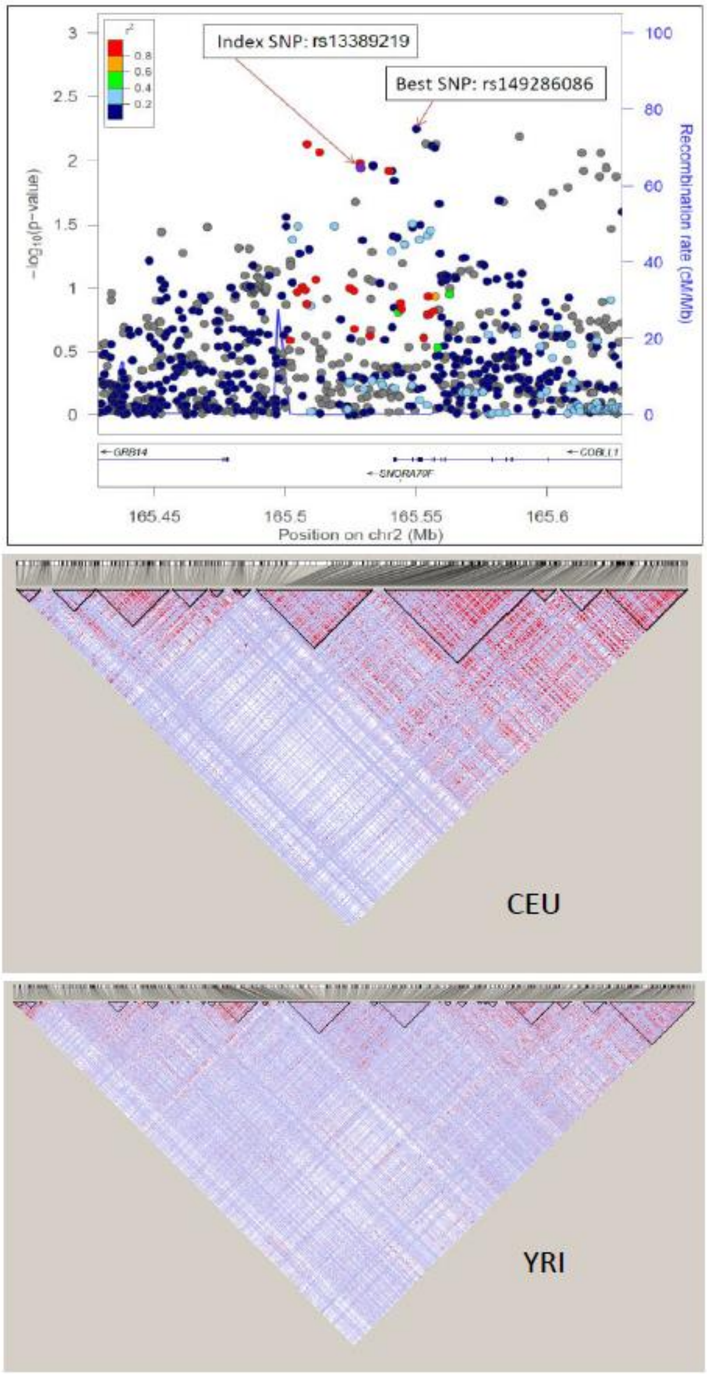

IGF2BP2

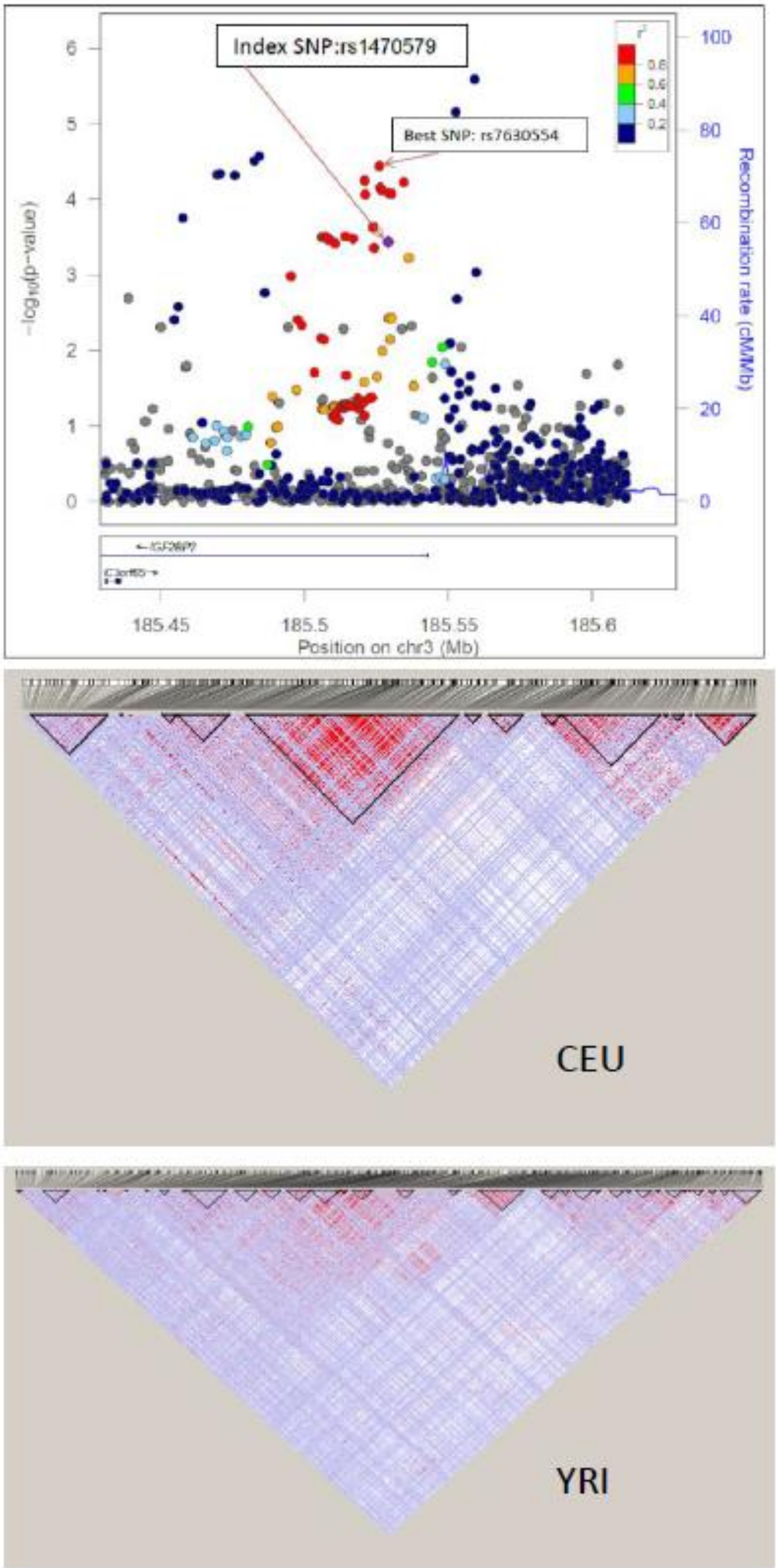

Index SNP:rs1470579
Best SNP: rs7630554
-log10(p-value)
Recombination rate (cM/Mb)
185.45
185.5
185.55
185.6
Position on chr3 (Mb)
CEU
YRI

ZBED3

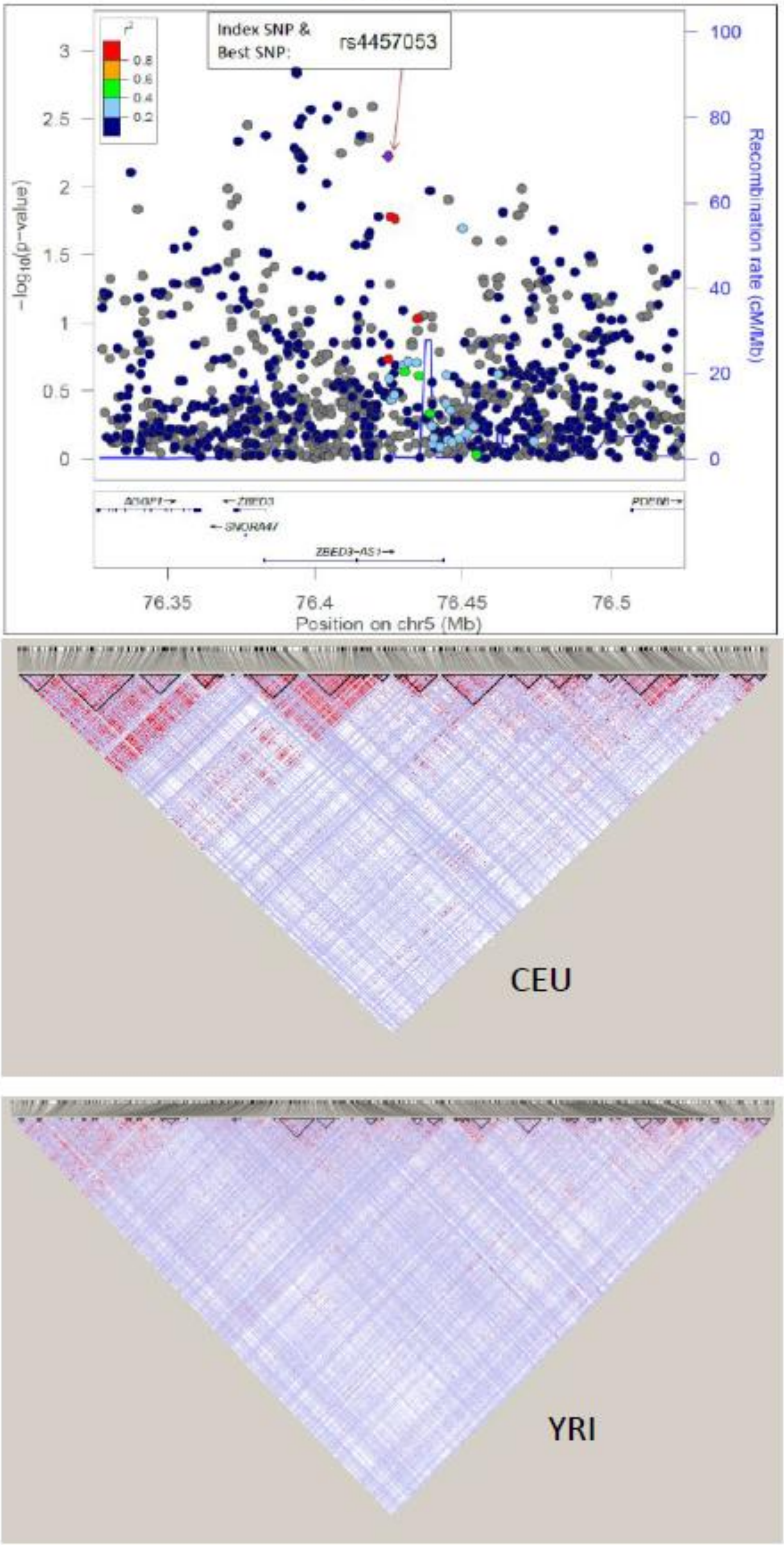
Index SNP &
Best SNP:
rs4457053
r^2
0.8
0.6
0.4
0.2
$-\log_{10}$(p-value)
Recombination rate (cM/Mb)
Position on chr5 (Mb)
76.35
76.4
76.45
76.5
ZBED3
SNORA47
ZBED3-AS1
PDE8B
CEU
YRI

CDKAL1

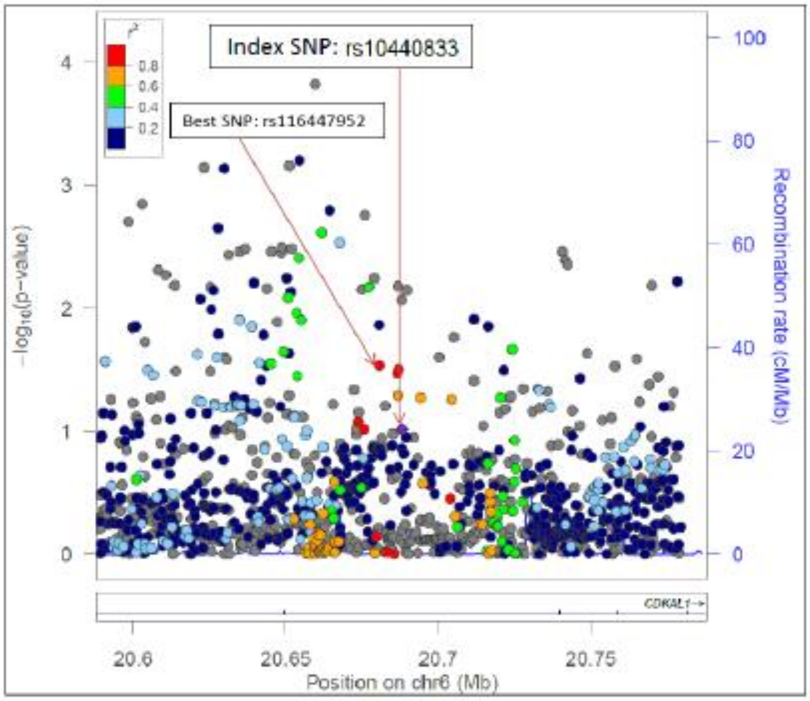

Index SNP: rs10440833
Best SNP: rs116447952
r^2
0.8
0.6
0.4
0.2
$-\log_{10}$(p-value)
Recombination rate (cM/Mb)
CDKAL1→
20.6
20.65
20.7
20.75
Position on chr6 (Mb)

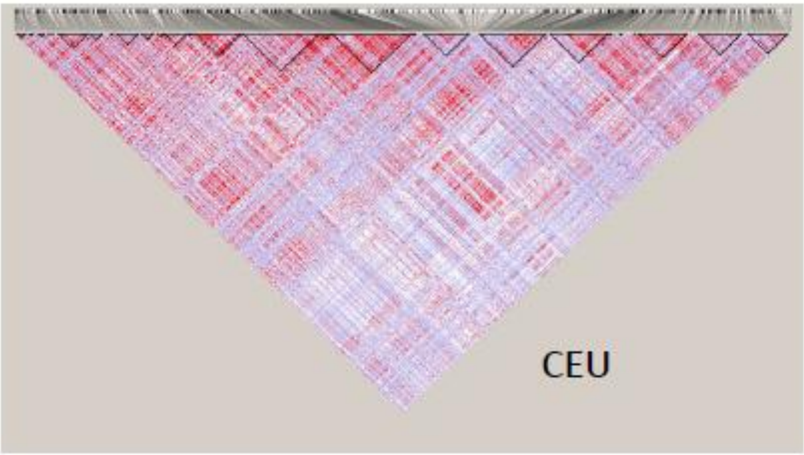

CEU

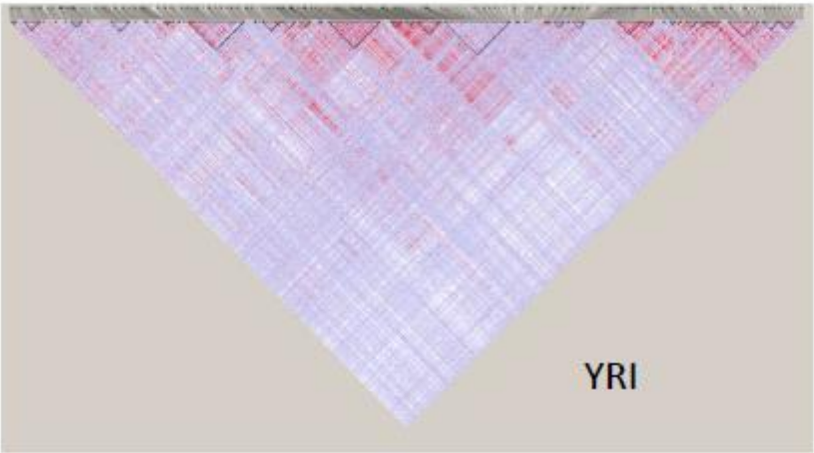

YRI

KLF14

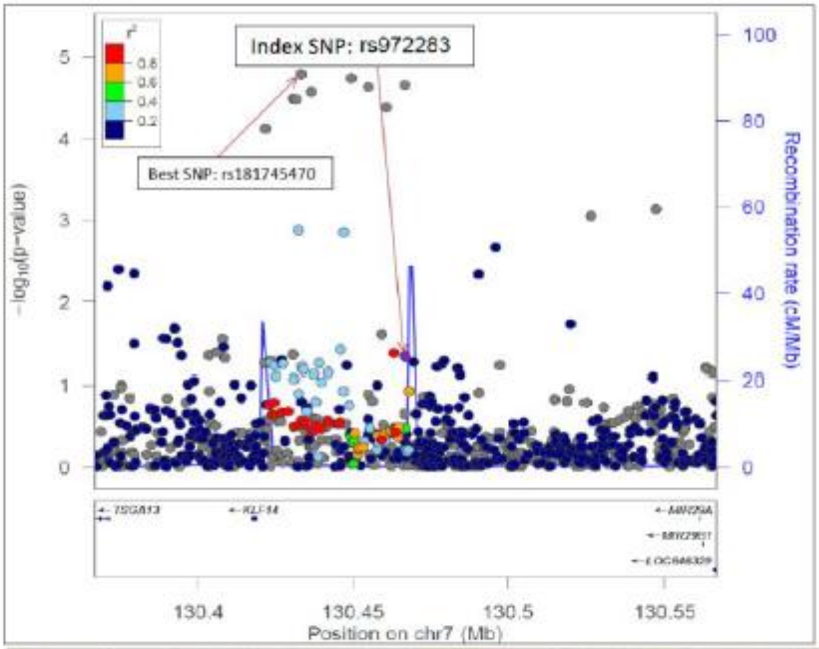
Index SNP: rs972283
Best SNP: rs181745470
r²
0.8
0.6
0.4
0.2
−log10(p-value)
Recombination rate (cM/Mb)
Position on chr7 (Mb)
130.4
130.45
130.5
130.55
KLF14

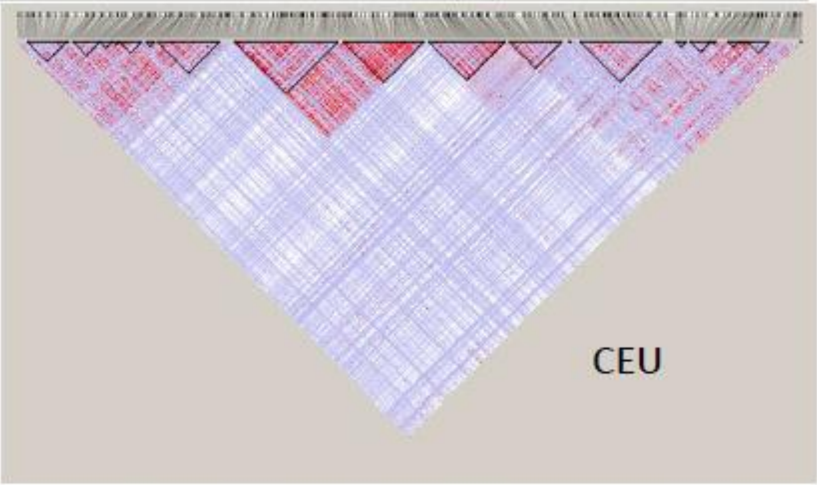
CEU

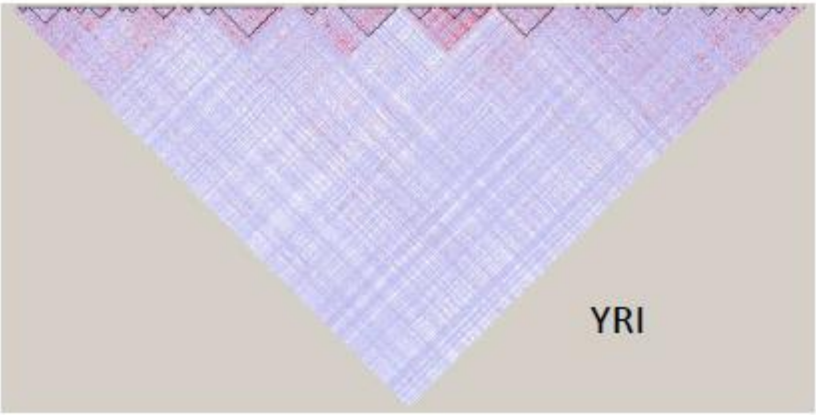
YRI

SLC30A8

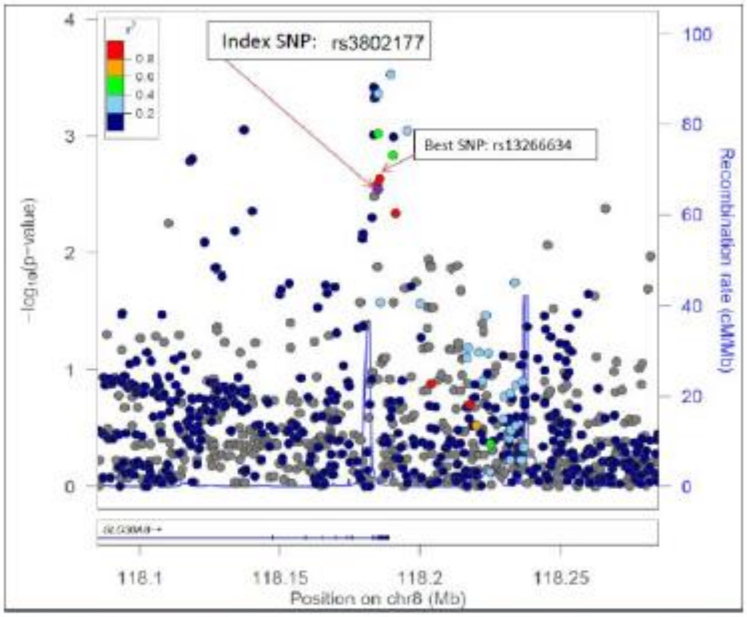

Index SNP: rs3802177
Best SNP: rs13266634
0.8
0.6
0.4
0.2
4
3
2
1
0
−log10(p−value)
100
80
60
40
20
0
Recombination rate (cM/Mb)
118.1
118.15
118.2
118.25
Position on chr8 (Mb)

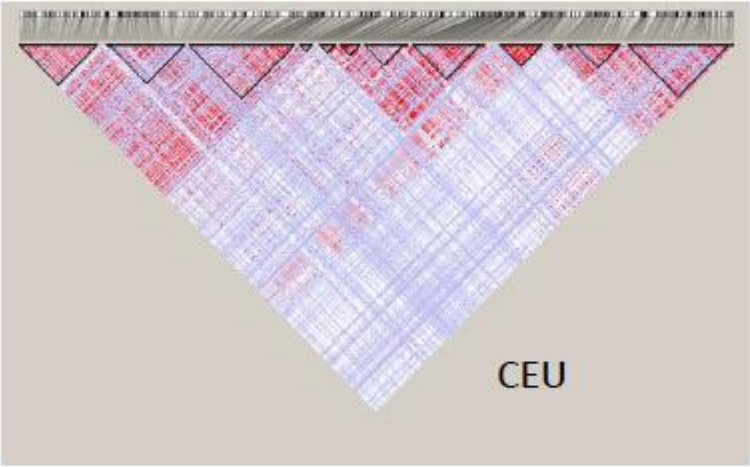

CEU

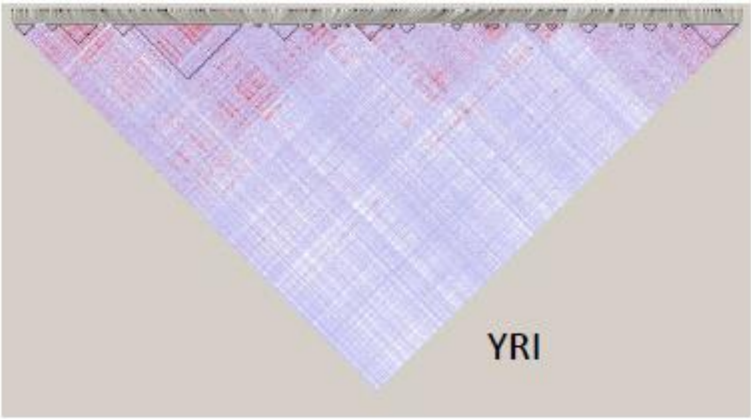

YRI

TCF7L2

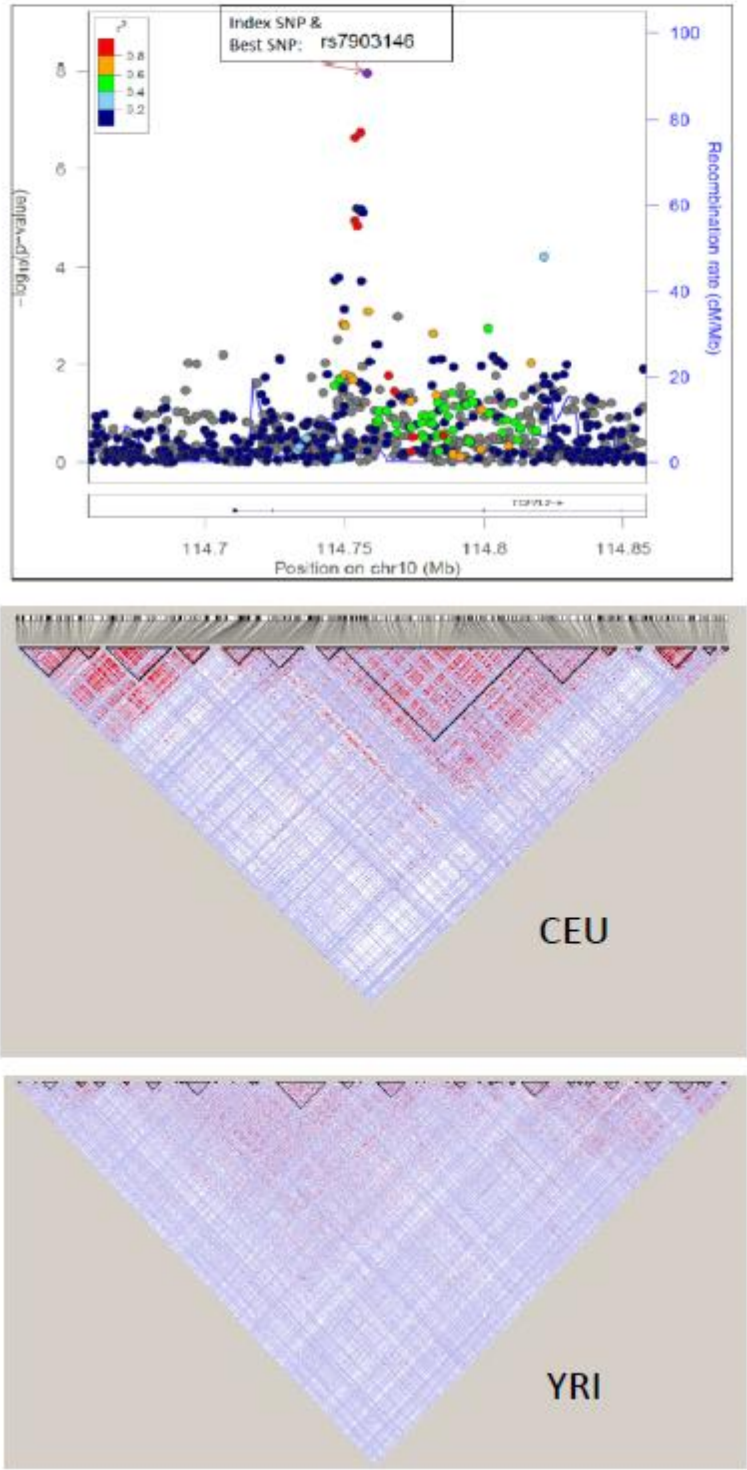
Index SNP &
Best SNP: rs7903146
r²
0.8
0.6
0.4
0.2
-log10(P-value)
Recombination rate (cM/Mb)
Position on chr10 (Mb)
114.7
114.75
114.8
114.85
CEU
YRI

HIGD1C

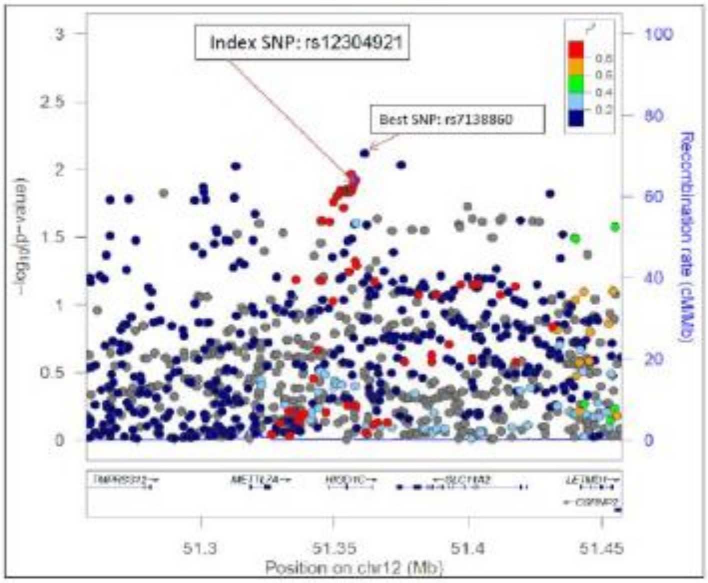
Index SNP: rs12304921
Best SNP: rs7138860
r²
0.8
0.6
0.4
0.2
3
2.5
2
1.5
1
0.5
0
−log10(p-value)
100
80
60
40
20
0
Recombination rate (cM/Mb)
51.3
51.35
51.4
51.45
Position on chr12 (Mb)

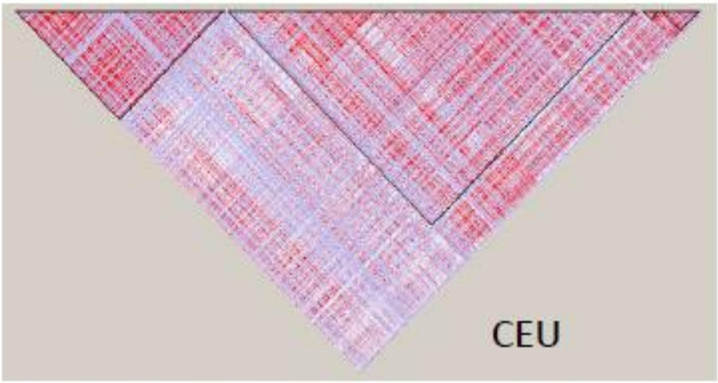
CEU

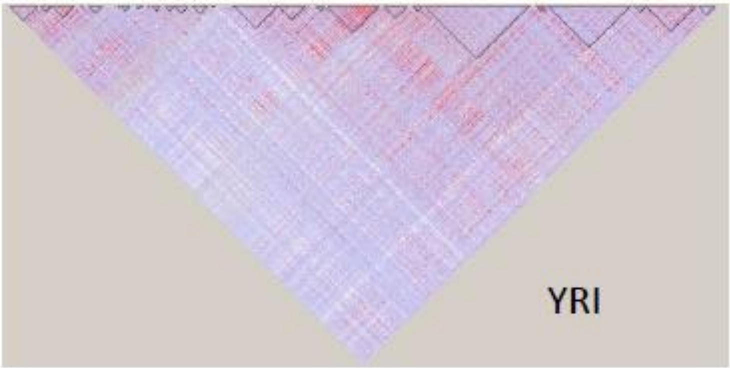
YRI

HMG20A

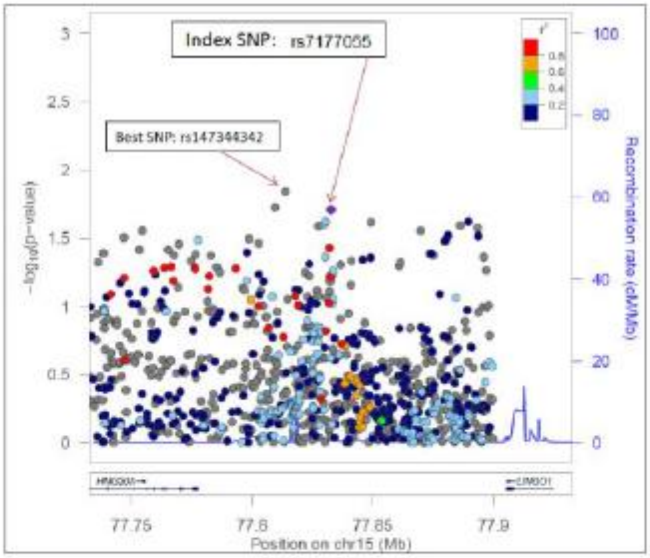
Index SNP: rs7177055
Best SNP: rs147344342
−log10(p−value)
Recombination rate (cM/Mb)
Position on chr15 (Mb)
77.75
77.8
77.85
77.9
0
0.5
1
1.5
2
2.5
3
0
20
40
60
80
100

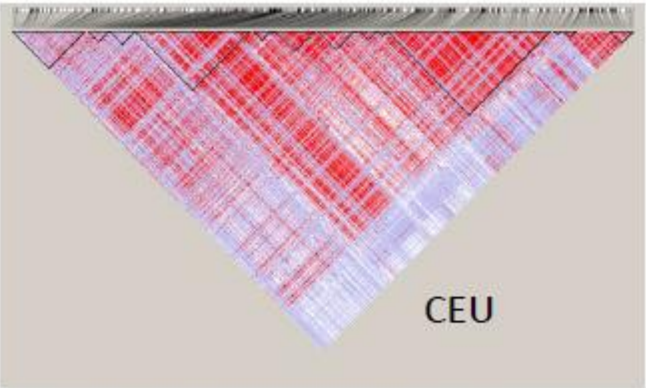
CEU

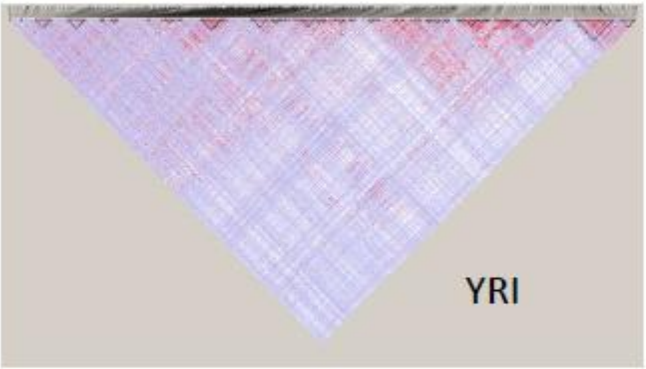
YRI

FTO

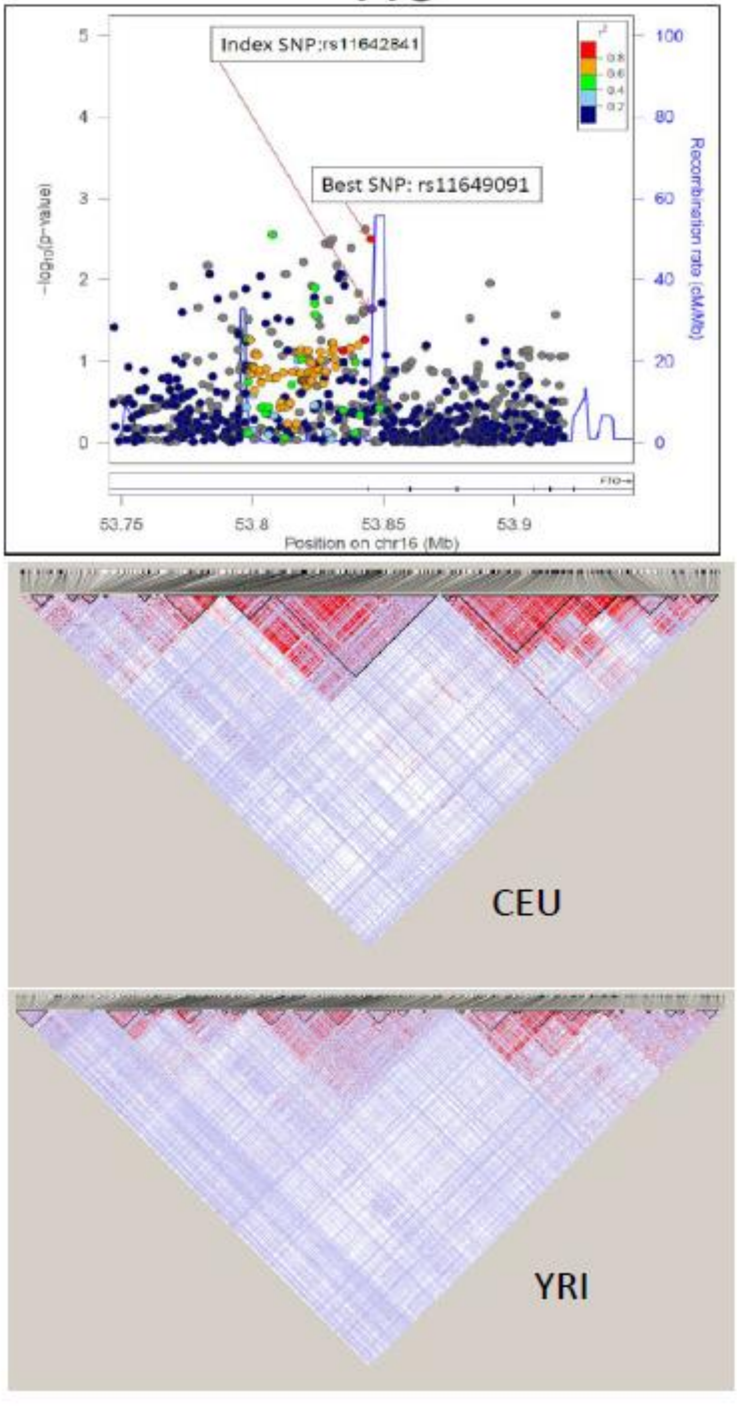

Index SNP:rs11642841
Best SNP: rs11649091
-log10(p-value)
Recombination rate (cM/Mb)
0
1
2
3
4
5
0
20
40
60
80
100
53.75
53.8
53.85
53.9
Position on chr16 (Mb)
CEU
YRI

PEPD

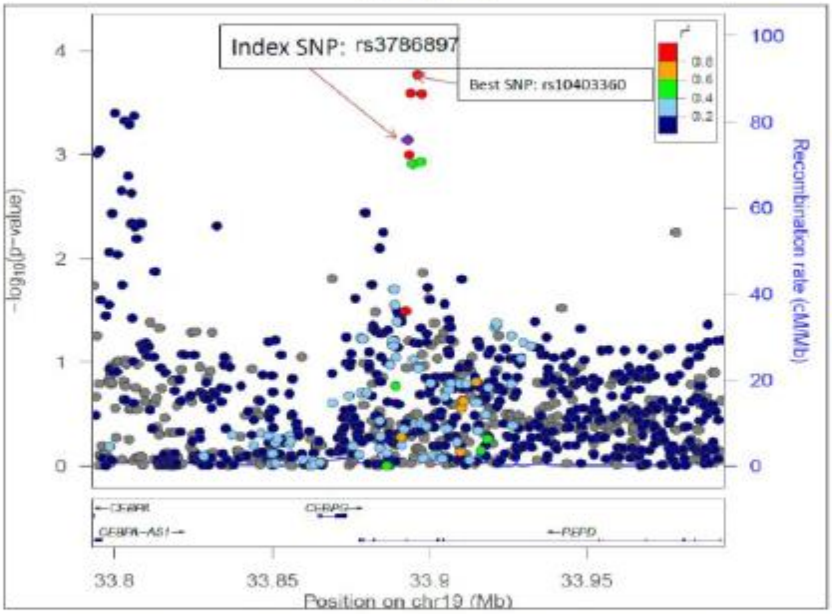
Index SNP: rs3786897
Best SNP: rs10403360
r²
0.8
0.6
0.4
0.2
−log10(p−value)
Recombination rate (cM/Mb)
Position on chr19 (Mb)
33.8
33.85
33.9
33.95
CEBPA
CEBPA−AS1
CEBPG
PEPD

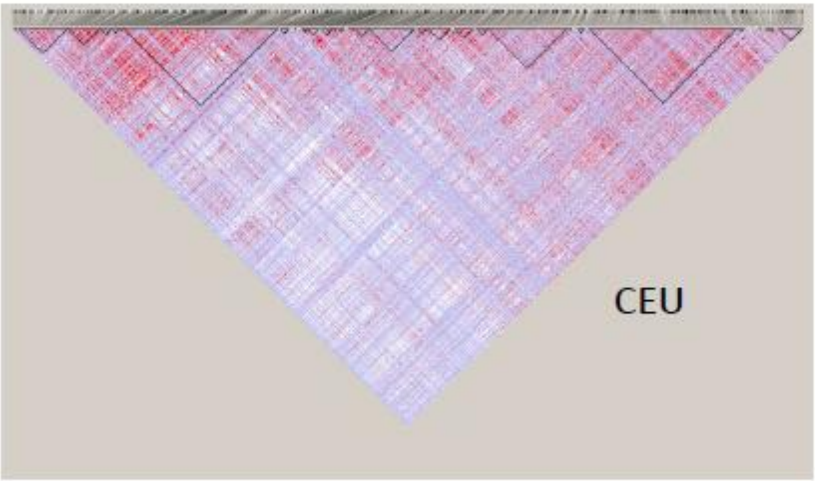
CEU

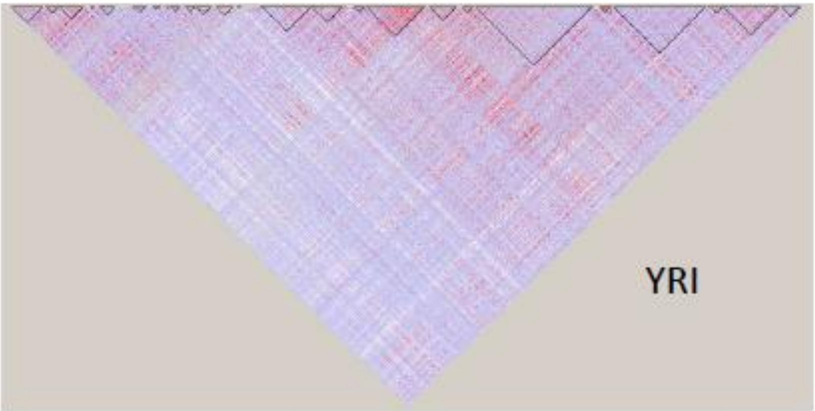
YRI
